# Long-term immune response to mRNA anti-SARS-CoV-2 vaccination in patients with cancer

**DOI:** 10.3389/fimmu.2026.1815933

**Published:** 2026-05-20

**Authors:** Soraia Lobo-Martins, Diogo Martins-Branco, Francine Padonou, Hafid Dahma, Sigi van den Wijngaert, Mounia Jaouart, Chiara Dauccia, Chiara Molinelli, Elisa Agostinetto, Angela Loizidou, Donatienne Taylor, Anais Boisson, Soizic Garaud, Chloé Spilleboudt, Jose Vicente Cardona, Peter Ramge, Bogdana Ioana Balas, Mohammed Bajji, Fanny George, Tabatha Delsaute, Marianne Paesmans, Lieveke Ameye, Martine Piccart, Evandro de Azambuja, Karen Willard-Gallo

**Affiliations:** 1 Academic Trials Promoting Team (ATPT), Hôpital Universitaire de Bruxelles (H.U.B), Institut Jules Bordet, Université Libre de Bruxelles (ULB), Brussels, Belgium; 2 Molecular Immunology Unit, Hôpital Universitaire de Bruxelles (H.U.B), Institut Jules Bordet, Université Libre de Bruxelles (ULB), Brussels, Belgium; 3 Laboratoire Hospitalier Universitaire de Bruxelles (LHUB-ULB), Université Libre de Bruxelles (ULB), Brussels, Belgium; 4 Department of Internal Medicine and Medical Therapy, University of Pavia, Pavia, Italy; 5 Department of Internal Medicine and Medical Specialties (DiMI), School of Medicine, Università di Genova, Genoa, Italy; 6 Department of Medical Oncology, UO Clinica di Oncologia Medica, IRCCS Ospedale Policlinico San Martino, Genoa, Italy; 7 Infectious Diseases Department, Hôpital Universitaire de Bruxelles (H.U.B), Institut Jules Bordet, Université Libre de Bruxelles (ULB), Brussels, Belgium; 8 Department of Medical Oncology, CHU UCL Namur/Site Sainte-Elisabeth, Namur, Belgium; 9 Hematology Department, Hôpital Universitaire de Bruxelles (H.U.B), Institut Jules Bordet, Université Libre de Bruxelles (ULB), Brussels, Belgium; 10 Pharmaceuticals Division – Global Product Development, Medical Affairs Oncology, F. Hoffmann-La Roche, Ltd., Basel, Switzerland; 11 Roche Diagnostics Solutions, Clinical Development and Medical Affairs (CDMA), Roche Diagnostics International Ltd., Rotkreuz, Switzerland; 12 Pharmaceuticals Division – Global Product Development, Clinical Safety Oncology, F. Hoffmann-La Roche, Ltd., Basel, Switzerland; 13 Clinical Trials Center (CTC), Hôpital Universitaire de Bruxelles (H.U.B), Institut Jules Bordet, Université Libre de Bruxelles (ULB), Brussels, Belgium; 14 Clinical Biostatistics Unit, Hôpital Universitaire de Bruxelles (H.U.B), Institut Jules Bordet, Université Libre de Bruxelles (ULB), Brussels, Belgium; 15 Hôpital Universitaire de Bruxelles (H.U.B), Institut Jules Bordet, Université Libre de Bruxelles (ULB), Brussels, Belgium; 16 Medical Oncology Department, Hôpital Universitaire de Bruxelles (H.U.B), Institut Jules Bordet, Université Libre de Bruxelles (ULB), Brussels, Belgium

**Keywords:** adaptive immunity, cancer immunology, humoral immune response, immune response determinants, mRNA vaccine immunogenicity, SARS-CoV-2 vaccination

## Abstract

**Introduction:**

Patients with cancer are at increased risk of morbidity and mortality from COVID-19 but were underrepresented in pivotal vaccine trials. Data on the magnitude and determinants of immune responses to mRNA SARS-CoV-2 vaccination in this population remain limited.

**Methods:**

I-SPARC is a prospective, phase IV clinical trial evaluating humoral and cellular immune responses to mRNA SARS-CoV-2 vaccination in 115 patients with cancer, including those receiving systemic therapy and those in remission. Anti-Spike antibody titers were measured longitudinally, and immunophenotyping was performed to assess T and B cell subsets. Clinical outcomes, including SARS-CoV-2 infection, were recorded.

**Results:**

All patients developed detectable anti-Spike antibodies, although absolute titers varied by cancer type and treatment. Patients with hematologic malignancies and/or receiving chemotherapy had the lowest anti-Spike antibody levels. Booster doses significantly increased titers, particularly in patients in remission or receiving non-cytotoxic therapies. Prior SARS-CoV-2 infection and the number of vaccine doses were associated with better responses. Immunophenotyping confirmed vaccine-induced expansion of memory T and B lymphocyte subpopulations. SARS-CoV-2 infection occurred in 16% of our cohort, with infrequent severe cases.

**Discussion:**

mRNA SARS-CoV-2 vaccines elicit robust humoral and cellular immune responses in patients with cancer, despite variability according to disease type and treatment. These findings support the use of booster strategies and provide a rationale for tailored vaccination approaches in immunocompromised populations.

## Introduction

The coronavirus disease 2019 (COVID-19) pandemic, caused by the severe acute respiratory syndrome coronavirus 2 (SARS-CoV-2), resulted in over 7 million deaths globally as of early 2025 (1). Multiple vaccines were rapidly developed, with mRNA-based platforms – such as Comirnaty^®^ (BNT162b2, Pfizer/BioNTech) and Spikevax^®^ (mRNA-1273, Moderna) – emerging as the cornerstone of worldwide vaccination strategies. These vaccines encode the SARS-CoV-2 spike glycoprotein, the principal target for both neutralizing antibodies and T-cell responses (2, 3). Phase III trials demonstrated that mRNA vaccines confer over 90% efficacy against symptomatic COVID-19 in the general population, with a favorable safety profile (4, 5). Their ability to elicit both humoral and cellular immunity underlies their robust protection, particularly against severe disease, and their prioritization in national and global vaccination programs.

Patients with cancer are particularly vulnerable to SARS-CoV-2 infection and ensuing complications. A pooled analysis of 52 studies, including 18, 650 patients, reported a mortality rate of 25.6%, markedly among those with lung or hematological malignancies (6). This heightened risk reflects a combination of cancer-associated immune dysfunction and treatment-induced immunosuppression. Patients with cancer were however excluded from pivotal vaccine trials, creating critical gaps relating to vaccine efficacy and safety in this population.

Patients with cancer have consistently attenuated immune responses to anti-SARS-CoV-2 vaccination, with substantial variability by tumor type, treatment modality and host-related factors. A systematic review reported lower seroconversion in patients with cancer versus healthy controls (73% vs. 94%), most notable in those receiving chemotherapy or anti-CD20 agents (7). A meta-analysis from our group, including 30, 183 patients across 89 studies, reported an overall seroconversion rate of 80% (94% in solid and 60% in hematological malignancies) (8). Cellular responses were observed in 61%, but more frequent in solid (68%) compared to hematological malignancies (59%). Subgroup analyses further identified older age, male sex, lymphopenia, and recent chemotherapy as predictors of weaker immune responses (9–11). Most published studies have focused on short-term outcomes following primary vaccination, were retrospective in design, and seldom included patients in remission – who can remain immunocompromised for long periods after treatment completion.

A few prospective trials have addressed early vaccine immunogenicity in patients with cancer although important uncertainties remain. The VOICE trial, a non-inferiority study, found that patients receiving chemotherapy, immunotherapy or both developed antibody responses after two doses of mRNA-1273, which was comparable to healthy controls (12). Nearly 20% of patients receiving chemotherapy however were suboptimal responders (defined *post hoc* as <300 binding antibody units/mL) with patients in remission excluded and a limited follow-up beyond three to four months. The CoviGI study evaluated humoral and cellular responses in patients with solid and hematological malignancies receiving systemic therapy (13). It demonstrated that while all patients with solid tumors ultimately seroconverted following BNT162b2 mRNA vaccination, both humoral and cellular immune responses were significantly impaired in those receiving chemotherapy. Together, these studies underscore the heterogeneity of immune responses in patients with cancer but still leave important gaps in the literature. There is limited data on immunological memory beyond six months, booster responses and immunogenicity in patients in remission. Few trials have prospectively evaluated both humoral and cellular immunity across treatment settings and longitudinal timepoints.

The objective of the I-SPARC clinical trial (NCT05075538; EudraCT 2021-003710-39) was to conduct a comprehensive evaluation of both humoral and cellular immune responses in patients with cancer following anti-SARS-CoV-2 mRNA vaccination. The aims of this study were two-fold: first, to assess immune durability and vaccine efficacy in patients actively receiving treatment as well as those in remission; and second, to address critical gaps in our knowledge to enhance future vaccination strategies for immunocompromised populations.

## Results

### Study population and baseline clinical characteristics

**Figure 1:**
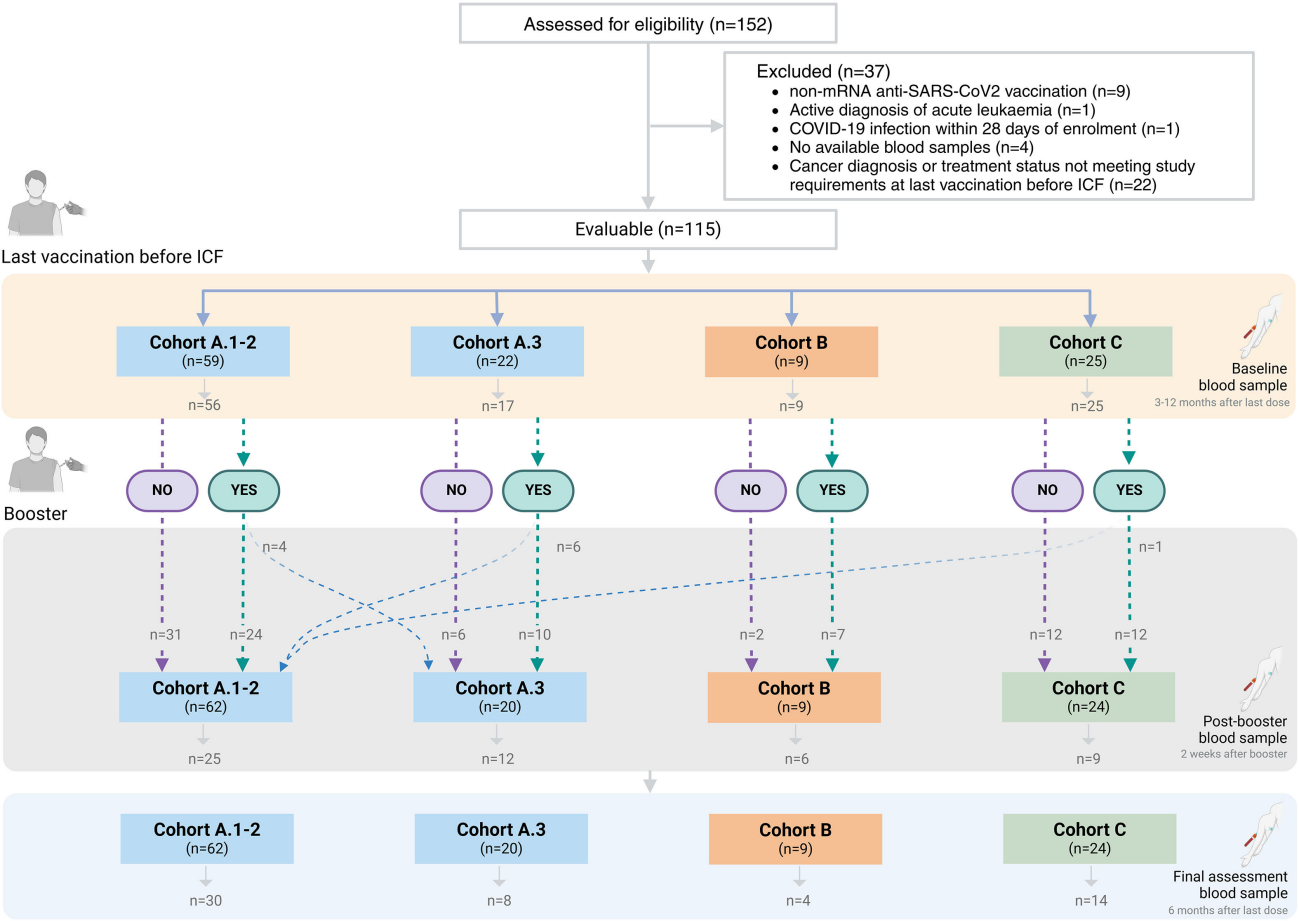
Patient flowchart and sample collection. Flowchart illustrating patient enrollment, cohort allocation, and number of patients with blood sample collection at each study assessment per study cohort. Patients who modified their treatment regimen were reassigned to a different cohort at the booster phase, indicated by dashed lines (for additional details see **Supplementary Figure 2**).

A total of 152 patients were enrolled between December 1, 2021 and October 31, 2023 with 115 evaluable patients, defined as patients contributing with at least one immunological assessment meeting cohort-specific criteria at any study timepoint (**Figure 1****;** **Supplementary Figure 1****;** **Supplementary Annex I**). Patients were categorized into cohort A.1–2 (N = 59, received immunotherapy, targeted agents or endocrine therapy without cytotoxic chemotherapy), cohort A.3 (N = 22, received cytotoxic chemotherapy, either alone or in combination with other treatments), cohort B (N = 9, hematologic malignancies receiving systemic treatment) or cohort C (N = 25, were in complete remission without any systemic cancer treatment for at least one year). Evaluability was assessed separately for each study timepoint; consequently, some patients contributed with evaluable samples only at post-booster or final assessments (**Supplementary Figure 1**; **Annex I**). Eleven patients switched cohorts between the prime and booster doses due to treatment alterations during the study follow-up (**Figure 1**; **Supplementary Figure 2**).

Baseline assessments (3–12 months after the last vaccine dose administered prior to study enrolment) were available for 107 patients (93%; 56 in cohort A.1–2, 17 in cohort A.3, 9 in cohort B, and 25 in cohort C), while post-boost assessments (within 2 weeks of a booster dose) were performed for 52 patients (45%; 25 in cohort A.1–2, 12 in cohort A.3, 6 in cohort B, and 9 in cohort C). Finally, 56 patients completed a final assessment (6 months ± 4 weeks after the baseline assessment or 6 months ± 4 weeks after a booster dose if administered during the study; 49%; 30 in cohort A.1–2, 8 in cohort A.3, 4 in cohort B, and 14 in cohort C). The median time between booster administration and final assessment was 174 days (interquartile range [IQR], 152–189). In patients who did not receive a booster during the study, the median time between baseline and final assessment was 188 days (IQR, 168–203). Missing assessments were primarily due to missed visits, logistical constraints in sample collection, or technical issues during sample processing. No systematic pattern of missingness across cohorts was identified; therefore, we assume missing values to be independent of the observed and unobserved data – missing completely at random.

Baseline characteristics at enrollment are summarized in **Table 1**. The median age was 61 years (interquartile range [IQR], 53–68) and 83% were female, although the sex distribution varied across cohorts — most patients with hematologic malignancies (cohort B) were male. The most common tumor type was breast cancer (69%), followed by lung cancer (9%) and lymphoid malignancies (4%). Among patients with solid tumors, 40% had metastatic disease at enrollment; as expected, most patients in cohort C (in remission) had non-metastatic disease (91%). Across the entire study population, 97% had an Eastern Cooperative Oncology Group (ECOG) performance status of 0–1. Additional baseline characteristics included a median body mass index (BMI) of 25 kg/m² (IQR, 22–30) with 54% classified as overweight or obese. Smoking history was classified in patients as 16% current, 20% former and 64% never. Laboratory abnormalities at screening were infrequent, with 24% presenting with lymphopenia (ALC <1, 000/μL) and 1% hypogammaglobulinemia. Anti-SARS-CoV-2 vaccination history included 21% that received two mRNA vaccine doses, 38% three doses, 33% four doses and 8% five doses prior to study entry. At enrollment, 25% of patients had their most recent mRNA anti-SARS-CoV-2 vaccination more than nine months earlier. Details for individual cohort B patients, including hematologic malignancy subtypes, systemic therapies and sample availability, are summarized in **Supplementary Table 1**.

**Table 1:** Baseline characteristics at enrolment.

	Total (N = 115)	Cohort				p-value
		A.1–2 (N = 59)25	A.3 (N = 22)	B (N = 9)	C (N = 25)	
Age						
Median (IQR)	61 (53, 68)	62 (54, 68)	60.5 (50, 67)	63 (49, 73)	59 (54, 67)	0.767^a^
18–55 years, n (%)	38 (33)	19 (32)	8 (36)	3 (33)	8 (32)	0.738^b^
56–75 years, n (%)	69 (60)	36 (61)	13 (59)	4 (44)	16 (64)	
>75 years, n (%)	8 (7)	4 (7)	1 (5)	2 (22)	1 (4)	
Gender, n (%)						
Female	95 (83)	54 (92)	20 (91)	2 (22)	19 (76)	<0.001^b^
Male	20 (17)	5 (8)	2 (9)	7 (78)	6 (24)	
Race, n (%)						
White	111 (97)	58 (98)	19 (86)	9 (100)	25 (100)	0.109^b^
Black/African American	2 (2)	1 (2)	1 (5)	0 (0)	0 (0)	
Asian	2 (2)	0 (0)	2 (9)	0 (0)	0 (0)	
Type of cancer, n (%)						
Primary solid tumor location						
Anal Canal	1 (1)	1 (1.7)	0 (0)	–	0 (0)	<0.001^b^
Bladder	3 (3)	0 (0)	1 (5)	–	2 (8)	
Breast	79 (69)	50 (85)	13 (59)	–	16 (64)	
Colorectal	2 (2)	0 (0)	2 (9)	–	0 (0)	
Head and Neck	1 (1)	0 (0)	0 (0)	–	1 (4)	
Lung	10 (9)	2 (3)	4 (18)	–	4 (16)	
Ovary	4 (3)	3 (5)	1 (5)	–	0 (0)	
Pancreas	1 (1)	0 (0)	1 (5)	–	0 (0)	
Prostate	3 (3)	3 (5)	0 (0)	–	0 (0)	
Hematological malignancy						
Leukemia	3 (3)	–	–	3 (33)	0 (0)	0.727^b^
Lymphoma	5 (4)	–	–	3 (33)	2 (8)	
Myeloma	2 (2)	–	–	2 (22)	0 (0)	
Other	1 (1)	–	–	1 (11)	0 (0)	
Cancer status at enrolment, n (%) ^c, d^						
Metastatic	41 (40)	26 (45)	13 (59)	NA	2 (9)	0.0006^b^
Non-metastatic	62 (60)	32 (55)	9 (41)	NA	21 (91)	
ECOG PS, n (%) ^e^						
0–1	111 (97)	57 (97)	21 (95)	8 (100)	25 (100)	0.822^b^
2	3 (3)	2 (3)	1 (5)	0 (0)	0 (0)	
BMI at screening ^f^						
Median (IQR)	25 (22, 30)	25 (23, 29)	25 (22, 28)	24 (22, 29)	27 (25, 32)	0.238^b^
Overweight/obese, n (%)	53 (54)	25 (50)	10 (50)	3 (50)	15 (68)	0.515^b^
Smoking status, n (%) ^g^						
Current	16 (16)	7 (14)	2 (11)	4 (50)	3 (14)	0.252^b^
Former	19 (20)	9 (18)	3 (17)	2 (25)	5 (23)	
Never	62 (64)	33 (67)	13 (72)	2 (25)	14 (64)	
ALC at screening (µL) ^h^						
Median (IQR)	1 395 (1 010, 1 880)	1 430 (1 060, 1 900)	1 140 (790, 1 610)	1 160 (900, 1 810)	1 670 (1 280, 2 110)	0.159^a^
<1 000µL, n (%)	24 (24)	11 (22)	7 (37)	4 (44)	2 (10)	0.089^b^
≥1 000µL, n (%)	76 (76)	40 (78)	12 (63)	5 (56)	19 (90)	
Lymphopenia since last visit, n (%)						
No	114 (99)	58 (98)	22 (100)	9 (100)	25 (100)	1.000^b^
Yes	1 (1)	1 (2)	0 (0)	0 (0)	0 (0)	
Hypogammaglobulinemia since last visit, n (%)						
No	114 (99)	58 (98)	22 (100)	9 (100)	25 (100)	1.000^b^
Yes	1 (1)	1 (2)	0 (0)	0 (0)	0 (0)	
Number of prior mRNA anti-SARS-CoV-2 vaccination, n (%)						
2 doses	24 (21)	7 (12)	8 (36)	4 (44)	5 (20)	0.175^b^
3 doses	44 (38)	23 (39)	8 (36)	3 (33)	10 (40)	
4 doses	38 (33)	24 (41)	4 (18)	1 (11)	9 (36)	
5 doses	9 (8)	5 (8)	2 (9)	1 (11)	1 (4)	
Time since last vaccination prior to inclusion, n (%)						
<6 months	42 (37)	20 (34)	10 (46)	4 (44)	8 (32)	0.496^b^
6 to 9 months	44 (38)	23 (39)	8 (36)	1 (11)	12 (48)	
>9 months	29 (25)	16 (27)	4 (18)	4 (44)	5 (20)	

Statistical test applied: (a) Kruskal-Wallis test; (b) Fisher Exact test. Cases of unknown/missing data per variable: (c) 1 for cohort A.1–2; (e) 1 for cohort B; (f) 9 for cohort A.1–2, 2 for cohort A.3, 3 for cohort B, and 3 for cohort C; (g) 10 for cohort A.1–2, 4 for cohort A.3, 1 for cohort B and 3 for cohort C; (h) 8 for cohort A.1–2, 3 for cohort A.3 and 4 for cohort C. Notes: (d) Only for patients with solid tumors. ALC, absolute lymphocyte count; BMI, body mass index; ECOG PS, Eastern Cooperative Oncology Group performance status; IQR, interquartile range; N, number; NA, not applicable; SARS-CoV-2, Severe Acute Respiratory Syndrome Coronavirus 2; µL, microliters.

### Humoral immune responses

#### Seropositive rates

All evaluable patients (N = 107, 100%) had anti-Spike antibody titers above the seropositive threshold (≥0.8 U/mL) at baseline. Seropositivity was maintained through all subsequent timepoints, including the post-boost (N = 52, 100%) and final (N = 53, 100%) assessments, implying a durable antibody response for the duration of the study (**Table 2**).

**Table 2:** Humoral immune response - anti-spike antibodies in the overall population and across the different cohorts at different study time points.

	Total	Cohorts				p-value
		A.1–2	A.3	B	C	
Baseline	N=107	N=56	N=17	N=9	N=25	
Seropositive rate, N (%, 95% CI)	107 (100, 97-100)	56 (100, 94-100)	17 (100, 80-100)	9 (100, 66-100)	25 (100, 86-100)	
Median, U/mL (IQR)	9, 017 (2, 180, 26, 871)	11, 293 (3, 359, 26, 487)	3, 875 (202, 13, 863)	330 (107, 2, 888)	8, 828 (3, 785, 28, 582)	0.005^a^
Anti-Nucleocapsid status at baseline ^b^						
Non-reactive						
N (%)	50 (47)	22 (40)	9 (53)	7 (78)	12 (48)	0.197^c^
Median, U/mL (IQR)	2, 865 (700, 5, 618)	3, 182 (961, 8, 869)	3, 197 (202, 4, 766)	246 (52, 1, 699)	4, 534 (817, 7, 277)	0.017^a^
Reactive						
N (%)	56 (53)	33 (60)	8 (47)	2 (22)	13 (52)	0.197^c^
Median, U/mL (IQR)	20, 800 (10, 866, 41, 483)	19, 193 (11, 205, 42, 893)	20, 367 (341, 30, 524)	21, 127 (10, 526, 31, 728)	28, 582 (18, 766, 46, 633)	0.655^a^
Post-boost	N=52	N=25	N=12	N=6	N=9	
Seropositive rate, N (%, 95% CI)	52 (100, 93-100)	25 (100, 86-100)	12 (100, 74-100)	6 (100, 54-100)	9 (100, 66-100)	
Median, U/mL (IQR)	30 079 (13 669, 56 337)	35 239 (23 192, 64 098)	25 094 (3 666, 62 819)	1 504 (287, 21 182)	30 414 (9 722, 39 142)	0.012^a^
Anti-Nucleocapsid status at post-boost ^d^						
Non-reactive						
N (%)	31 (62)	14 (58)	6 (50)	4 (67)	7 (88)	0.387^c^
Median, U/mL (IQR)	20 503 (6 710, 33 563)	29 992 (19 394, 41 522)	3666 (380, 20 444)	1 504 (356, 11 883)	30 414 (8 699, 39 142)	0.007^a^
Reactive						
N (%)	19 (38)	10 (41.7)	6 (50)	2 (33)	1 (13)	0.387^c^
Median, U/mL (IQR)	48 396 (28 179, 76 371)	49 900 (35 162, 76 371)	62 819 (34 317, 91 697)	14 185 (191, 28 179)	29 401 (29 401, 29 401)	0.233^a^
Final study assessment	N=56	N=30	N=8	N=4	N=14	
Seropositive rate, N (%, 95% CI)	56 (100, 94-100)	30 (100, 88-100)	8 (100, 63-100)	4 (100, 40-100)	14 (100, 77-100)	
Median, U/mL (IQR)	10 024 (5 040, 25 814)	10 106 (5 302, 24 628)	7 720 (2 334, 14 041)	3 941 (1 193, 6 433)	13 659 (8 792, 31 475)	0.053^a^
Anti-Nucleocapsid status at final study assessment						
Non-reactive						
N (%)	23 (41)	15 (50)	2 (25)	2 (50)	4 (29)	0.433^c^
Median, U/mL (IQR)	5 302 (2 269, 9 287)	5 368 (2 016, 15 297)	2 053 (582, 3 524)	3 941 (2 269, 5 613)	6 684 (4 059, 9 040)	0.348^a^
Reactive						
N (%)	33 (59)	15 (50)	6 (75)	2 (50)	10 (71)	0.433^c^
Median, U/mL (IQR)	17 866 (8 967, 31 475)	24 628 (8 967, 33 961)	10 205 (5 244, 17 866)	3 685 (117, 7 253)	29 375 (13 238, 54 363)	0.047^a^

Statistical test applied: (a) Kruskal-Wallis test; (c) Fisher Exact test. Cases of unknown/missing data per variable: (b) 1 for cohort A.1–2; (d) 1 for cohort A.1–2, and 1 for cohort C. Notes: Anti-Spike and anti-Nucleocapsid positivity was defined as a value ≥ 0.80 U/mL, measured using the Elecsys^®^ Anti-SARS-CoV-2 immunoassay (Roche Diagnostics).IQR, interquartile range; N, number; SARS-CoV-2, Severe Acute Respiratory Syndrome Coronavirus 2; U/mL, units per milliliter.

### Absolute titers of anti-spike antibodies

#### Titers of anti-spike antibodies across timepoints

**Figure 2:**
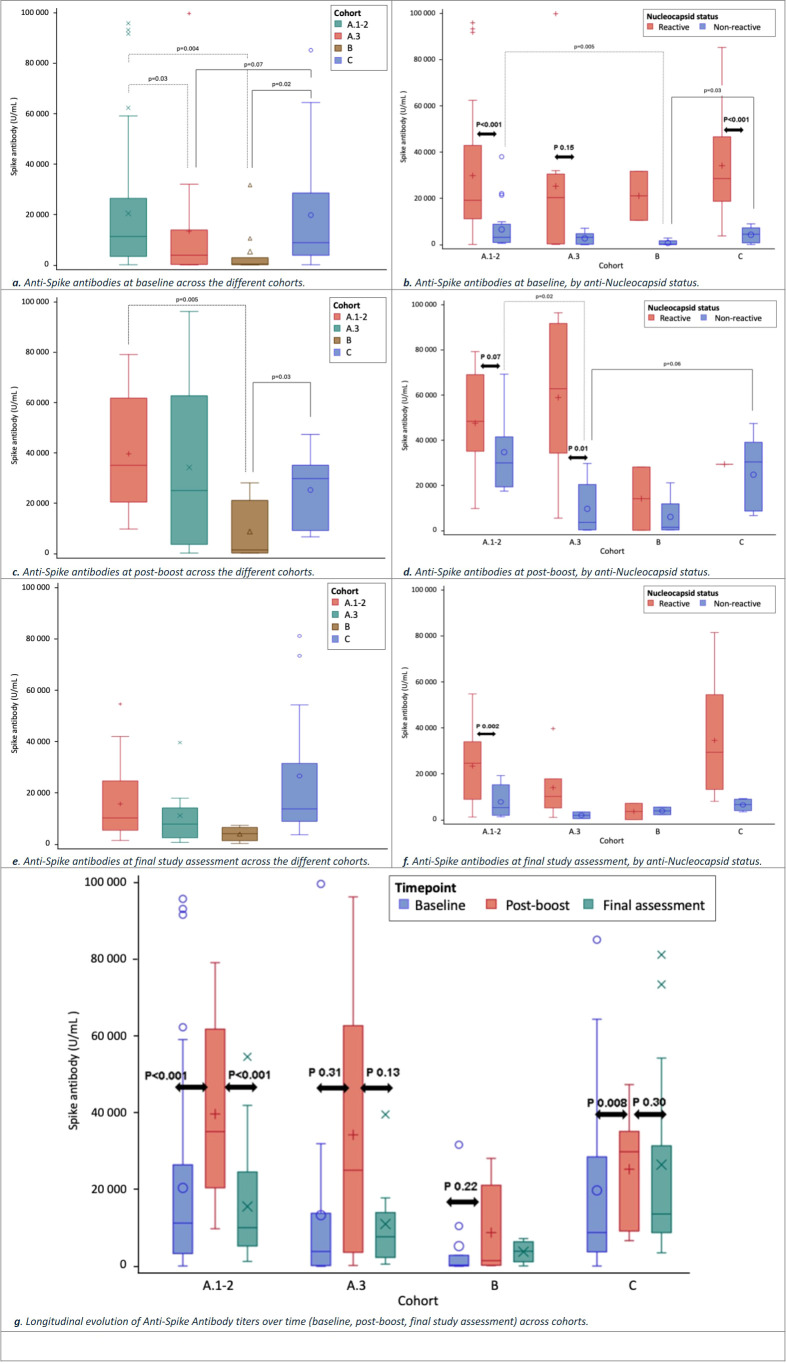
Anti-spike antibody titers across cohorts and timepoints. **(A)** Anti-Spike antibody titers at baseline across cohorts. **(B)** Anti-Spike antibody titers at baseline stratified by anti-SARS-CoV-2 nucleocapsid status. **(C)** Anti-Spike antibody titers at post-boost across cohorts. **(D)** Anti-Spike antibody titers at post-boost stratified by anti-SARS-CoV-2 nucleocapsid status. **(E)** Anti-Spike antibody titers at final study assessment across cohorts. **(F)** Anti-Spike antibody titers at final study assessment stratified by anti-SARS-CoV-2 nucleocapsid status. **(G)** Longitudinal evolution of anti-Spike antibody titers across cohorts from baseline to post-boost and final study assessment. Boxplots represent the distribution of anti-Spike antibody titers. Antibody levels were measured using the Elecsys® Anti-SARS-CoV-2 S assay (Roche Diagnostics) at predefined timepoints. Statistical comparisons were performed using the Mann–Whitney U test or Kruskal–Wallis test, as appropriate. Dotted lines indicate pairwise comparisons between cohort A.1–A.2 and other cohorts; solid lines indicate comparisons between cohort C and other cohorts. Double-sided arrows indicate within-cohort comparisons between anti-nucleocapsid reactive and non-reactive patients. P values are shown adjacent to the corresponding comparisons.

Absolute titers of anti-Spike antibodies varied significantly between cohorts and timepoints (**Table 2**; **Figure 2**). At baseline, the overall median anti-Spike antibody titer was 9, 017 U/mL [IQR, 2, 180–26, 871], with a significant difference observed across the cohorts (p=0.005). Patients in cohort A.3 exhibited lower titers (3, 875 U/mL [IQR, 202–13, 863]) compared to those in cohort A.1–2 (11, 293 U/mL [IQR, 3, 359–26, 487], p=0.003) and cohort C (8, 828 U/mL [IQR, 3, 785–28, 582], p=0.07). Patients in cohort B had the lowest titers (330 U/mL [IQR, 107–2, 888]), which were significantly lower than those in cohort A.1–2 (p=0.004) and cohort C (p=0.02) (**Table 2**; **Figure 2A**).

At the post-boost timepoint, the median anti-Spike antibody levels increased to 30, 079 U/mL [IQR, 13, 669–56, 337], with significant differences across the cohorts (p=0.012). Patients in cohort B again had the lowest titers (1, 504 U/mL [IQR, 287–21, 182]), which was significantly lower than cohort A.1–2 (35, 239 U/mL [IQR, 23, 192–64, 098], p=0.005) and cohort C (30, 414 U/mL [IQR, 9, 722–39, 142], p=0.03). Patients in cohort A.3 had numerically lower titers (25, 094 U/mL [IQR, 3, 666–62, 819]) than cohort A.1–2 and cohort C, although these differences were not statistically significant (p=0.18 and p=0.55, respectively) (**Table 2**, **Figure 2C**).

At the final assessment, the overall median anti-Spike antibody titer was 10, 024 U/mL [IQR, 5, 040–25, 814], with no statistically significant differences across the cohorts (p=0.053). Median titers remained numerically lower in patients in cohort A.3 (7, 720 U/mL [IQR, 2, 334–14, 041]) and cohort B (3, 941 U/mL [IQR, 1, 193–6, 433]) compared to cohort A.1–2 (10, 106 U/mL [IQR, 5, 302–24, 628]) and cohort C (13, 659 U/mL [IQR, 8, 792–31, 475]) (**Table 2**; **Figure 2E**).

Anti-Spike antibody titers at the pre-boost timepoint were quantified and are available in supplementary data (**Supplementary Table 2**). This analysis was only performed for a small subset of patients whose booster injections were scheduled more than two weeks after the baseline visit. In compliance with the study protocol, a separate pre-boost blood sample was not collected in patients that received their booster within 14 days from baseline, as this sample was considered representative of the pre-boost timepoint.

#### Longitudinal changes in anti-spike antibody titers within each cohort

Anti-Spike antibody titers fluctuated over time, with distinct patterns observed across all cohorts (**Figure 2G**), reflected by increased titers post-boost in all cohorts. The increase was statistically significant for cohort A.1–2 (from 11, 293 U/mL to 35, 239 U/mL [IQR, 23, 192–64, 098], p<0.001) and cohort C (from 8, 828 U/mL to 30, 414 U/mL [IQR, 9, 722–39, 142], p=0.008). In contrast, the increases observed in cohort A.3 (from 3, 875 U/mL to 25, 094 U/mL [IQR, 3, 666–62, 819], p=0.31) and cohort B (from 330 U/mL to 1, 504 U/mL [IQR, 287–21, 182], p=0.22) did not reach statistical significance (**Figure 2G**).

At the final assessment, anti-Spike antibody titers decreased significantly in cohort A.1–2 (from 35, 239 U/mL post-boost to 10, 106 U/mL [IQR, 5, 302–24, 628], p<0.001). A numerical decrease was also observed in cohort A.3 (to 7, 720 U/mL [IQR, 2, 334–14, 041], p=0.13) and cohort C (to 13, 659 U/mL [IQR, 8, 792–31, 475], p=0.30). Conversely, in cohort B, a numerical increase in titers was observed (to 3, 941 U/mL [IQR, 1, 193–6, 433] but statistical tests were not performed due to the small sample size (n<5)) (**Figure 2G**).

There was no statistical difference between the final and baseline assessments across all cohorts (all p>0.30; formal statistical testing was not performed for cohort B due to limited paired samples – **Supplementary Table 3**).

Individual patient-level trajectories of anti-Spike antibody titers over time are shown in **Supplementary Figures 3**-**S6** for the individual cohorts.

#### Impact of the anti-nucleocapsid antibody status on anti-spike antibody titers

Anti-Spike antibody titers varied based on the anti-Nucleocapsid antibody status (reactive vs. non-reactive), with lower levels of anti-Spike antibody consistently observed in patients with non-reactive anti-Nucleocapsid antibodies across cohorts and timepoints (**Table 2**; **Figure 2**). In cohort A.1–2, anti-Spike titers were significantly lower in non-reactive compared to reactive patients at baseline (3, 182 U/mL [IQR, 961–8, 869] vs. 19, 193 U/mL [IQR, 11, 205–42, 893], p<0.001) and at the final assessment (5, 368 U/mL [IQR, 2, 016–15, 297] vs. 24, 628 U/mL [IQR, 8, 967–33, 961], p=0.002), while the difference was not statistically significant at post-boost (29, 992 U/mL [IQR, 19, 394–41, 522] vs. 49, 900 U/mL [IQR, 35, 162–76, 371], p=0.070) (**Table 2**; **Figure 2**). In cohort A.3, non-reactive participants had numerically lower anti-Spike titers at baseline (3, 197 U/mL [IQR, 202–4, 766] vs. 20, 367 U/mL [IQR, 341–30, 524], p=0.149) and at final assessment (2, 053 U/mL [IQR, 582–3, 524] vs. 10, 205 U/mL [IQR, 5, 244–17, 866]; statistical test not performed due to n<5). A statistically significant difference was observed at post-boost (3, 666 U/mL [IQR, 380–20, 444] vs. 62, 819 U/mL [IQR, 34, 317–91, 697], p=0.010) (**Table 2**, **Figure 2**). In cohort B, statistical tests were not performed at any timepoint due to the small sample size (n<5). In cohort C, anti-Spike titers were significantly lower in non-reactive patients at baseline (4, 534 U/mL [IQR, 817–7, 277] vs. 28, 582 U/mL [IQR, 18, 766–46, 633], p<0.001), while post-boost and final assessments were not analyzed due to the limited patient numbers (n<5) (**Table 2**; **Figure 2**).

Among non-reactive individuals, pairwise comparisons at baseline showed that anti-Spike titers in cohort B were significantly lower than in cohorts A.1–2 and C (p= 0.005 and p=0.03, respectively), while titers in cohort A.3 did not differ significantly from A.1–2 or C (p=0.28 and p=0.20, respectively). At post-boost, anti-Spike titers in cohort A.3 were significantly lower than cohorts A.1–2 (p=0.02) and showed a non-significant trend toward lower values compared to cohort C (p=0.06). At final assessment, anti-Spike antibody titers remained numerically lower for those in cohort A.3 and B (statistical testing not performed due to small sample sizes (n<5)) (**Table 2**; **Figure 2**).

Among patients with reactive anti-Nucleocapsid status, cohort C had numerically higher anti-Spike antibody titers at baseline compared to the other cohorts (p=0.087). At post-boost, anti-Spike titers in cohort B were numerically lower than in the other cohorts (p=0.092). At final assessment, anti-Spike titers in cohorts A.3 and B were numerically lower than the other cohorts (**Table 2**; **Figure 2**).

Additional comparisons by anti-Nucleocapsid antibody status at baseline, post-boost, and the final study timepoints are shown in **Supplementary Tables 4**–**14**.

#### Anti-spike antibody titers between the clinical and vaccination subgroups

Anti-Spike antibody titers varied across the clinical and demographic subgroups (**Table 3**). All subgroup analyses in this section are exploratory and were not adjusted for multiple testing. Age was not associated with significant differences in anti-Spike antibodies titers at any timepoint (tested as categorical and continuous variables). Female patients had numerically higher median anti-Spike antibody titers compared to males, particularly at baseline (9, 227 U/mL [IQR, 3, 126–27, 727] vs. 2, 888 U/mL [IQR, 246–22, 468], p=0.069) and post-boost (30, 414 U/mL [IQR, 17, 677–51, 403] vs. 9, 722 U/mL [IQR, 424–61, 271], p=0.222) evaluations. Body mass index (BMI) was associated with differences in anti-Spike antibody titers post-boost, with overweight or obese patients showing significantly higher titers (32, 808 U/mL [IQR, 23, 192–69, 259]) compared to those with a BMI <25 Kg/m2 (19, 394 U/mL [IQR, 5, 572–35, 239], p=0.030). No significant differences were observed at baseline or final assessment (p=0.197 and p=0.392, respectively). No meaningful differences in anti-Spike antibody titers were detected based on smoking status, absolute lymphocyte counts (ALC) at screening, cancer status (metastatic vs. non-metastatic), lymphopenia or hypogammaglobulinemia (**Table 3**).

**Table 3:** Anti-spike antibodies and subgroup analysis.

	Baseline			Post-boost			Final study		
	N	Median, U/mL (IQR)	p-value	N	Median, U/mL (IQR)	p-value	N	Median, U/mL (IQR)	p-value
Nucleocapsid status ^a^									
Non-reactive	50	2 865 (700, 5 618)	<0.001 ^b^	31	20 503 (6 710, 33 563)	0.003 ^b^	23	5 302 (2 269, 9 287)	<0.001 ^b^
Reactive	56	20 800 (10 866, 41 483)		19	48 396 (28 179, 76 371)		33	17 866 (8 967, 31 475)	
Age ^c^									
18–55 years	33	7 074 (776, 15 479)	0.196 ^b^	17	33 563 (17 677, 48 396)	0.892 ^b^	17	5 866 (3 524, 18 292)	0.455 ^b^
56–75 years	66	9 426 (2 888, 29 032)		33	29 401 (17 515, 61 271)		37	10 359 (5 368, 28 977)	
>75 years	8	12 623 (2 974, 34 869)		2	26 993 (2583, 51403)		2	8 085 (7 253, 8 917)	
Sex									
Female	88	9 227 (3 126, 27 727)	0.069 ^b^	43	30 414 (17 677, 51 403)	0.222 ^b^	46	10 287 (5 244, 24 628)	0.535 ^b^
Male	19	2 888 (246, 22 468)		9	9 722 (424, 61 271)		10	7 680 (4 576, 27 725)	
BMI ^d^									
Overweight or obese	49	9 331 (3 054, 31 728)	0.197 ^b^	26	32 808 (23 192, 69 259)	0.030 ^b^	33	10 359 (4 836, 26 999)	0.392 ^b^
Other	42	9 058 (2 180, 16 112)		15	19 394 (5 572, 35 239)		15	8 917 (1 693, 24 628)	
Smoking status ^e^									
Current	16	8 598 (2 294, 14 860)	0.640 ^b^	10	22 187 (19 394, 28 179)	0.477 ^b^	6	8 697 (5 613, 10 359)	0.252 ^b^
Former	18	10 560 (556, 22 273)		11	48 396 (5 572, 69 259)		9	4 836 (3 541, 18 292)	
Never	57	9520 (3 310, 31 728)		26	30 079 (9 822, 35 239)		33	13 238 (5 368, 29 120)	
ALC at screening ^f^									
<1 000µL	21	9 098 (2 359, 21 402)	0.520 ^b^	8	29 326 (1 504, 43 538)	0.366 ^b^	6	4 384 (2 016, 10 215)	0.519 ^b^
≥1 000µL	72	9 717 (3 182, 28 807)		6	35 201 (28 041, 61 899)		8	7 904 (3 265, 23 433)	
Status at baseline ^g^									
Non-metastatic	56	9 425 (2 904, 31 241)	0.607 ^b^	28	33 940 (19 090, 54 834)	0.963 ^b^	33	13 238 (5 368, 31 024)	0.250 ^b^
Metastatic	39	9 098 (3 054, 22 468)		17	30 473 (19 394, 64 366)		19	9 852 (5 244, 17 866)	
Lymphopenia since last visit									
No	–	–	–	38	29 628 (9 822, 61 271)	0.885 ^b^	42	10 364 (5 603, 27 725)	0.100 ^b^
Yes	–	–		14	32 818 (17 677, 51 403)		14	5 429 (2 016, 17 745)	
Hypogammaglobulinemia since last visit									
No	–	–	–	49	29 744 (9 822, 61 271)	–	51	10 195 (5 302, 26 999)	0.446 ^b^
Yes	–	–		3	30 473 (28 041, 35 239)		5	4 836 (3 524, 17 745)	
No of prior doses									
2-3	61	3, 785 (857, 15, 479)	0.001 ^b^	45	30, 473 (17, 591, 51, 403)	0.639 ^b^	34	10, 364 (4, 836, 29, 120)	0.568 ^b^
4-5	46	15, 954 (7, 115, 29, 034)		7	20, 444 (5, 572, 91, 697)		22	8, 955 (5, 244, 18, 292)	
Time since last mRNA anti-SARS-CoV-2 vaccination dose before enrolment									
<6 months	38	4, 321 (756, 16, 238)	0.077 ^b^	NA	NA	–	NA	NA	–
6–9 months	42	13, 093 (3, 407, 29, 034)		NA	NA		NA	NA	
>9 months	27	9, 017 (3, 197, 26, 871)		NA	NA		NA	NA	

Statistical test applied: (b) Kruskal-Wallis test. (c) When analyzed as a continuous variable, age was not significantly associated with anti-Spike antibody titers at any timepoint (Spearman correlation: p = 0.210, 0.146, and 0.590 for baseline, post-boost, and final assessments, respectively). Cases of unknown/missing data per variable: (a) 1 for baseline, 2 at post-boost; (d) 16 for baseline, 11 at post-boost and 8 at final study assessment; (e) 16 for baseline, 5 for post-boost and 8 for final study assessment; (f) 14 for baseline, 38 for post-boost and 42 for final study assessment. Notes: (g) Only for patients with solid tumors. Anti-Spike and anti-Nucleocapsid positivity were defined as a value ≥ 0.80 U/mL, measured using the Elecsys^®^ Anti-SARS-CoV-2 immunoassay (Roche Diagnostics).ALC, absolute lymphocyte count; BMI, body mass index; ECOG PS, Eastern Cooperative Oncology Group performance status; IQR, interquartile range; N, number; NA, non-applicable; SARS-CoV-2, Severe Acute Respiratory Syndrome Coronavirus 2; U/mL, unit per milliliter; µL, microliters.

Interestingly, vaccination-related factors were associated with anti-Spike antibody titers (**Table 3**). Not unexpectedly, the number of prior anti-SARS-CoV-2 vaccinations was significantly associated with anti-Spike antibody titers at baseline, with higher median titers for patients with 4–5 doses (15, 954 U/mL [IQR, 7, 115–29, 034]) compared to those with 2–3 doses (3, 785 U/mL [IQR, 857–15, 479], p=0.001). This association however did not remain statistically significant at the post-boost or final assessment timepoints (p=0.639 and p=0.568, respectively). A trend towards higher baseline anti-Spike antibody titers was detected in patients who received their last vaccine dose between six to nine months compared to those who received it less than 6 months before enrollment, though no statistically significant associations were identified. When analyzed as a continuous variable, the interval between the last vaccination and baseline anti-Spike antibody titers was not significantly associated with antibody levels (Spearman ρ = 0.18, 95% CI −0.01 to 0.36; p = 0.063).

Subgroup analyses of anti-Spike antibody titers at the pre-boost timepoint based on age, sex, BMI, smoking status and lymphocyte counts are detailed in **Supplementary Tables 15**–**S19**.

#### Multivariable analysis of baseline anti-spike antibody titers

A multivariable robust regression analysis was performed to identify independent factors associated with baseline anti-Spike antibody titers (**Table 4**). We initially included clinical and demographic variables in the full model, including cohort, sex, cancer stage, timing since last vaccine dose (tested as categorical and continuous), number of prior vaccine doses, and anti-nucleocapsid status. Using backward variable selection, only anti-nucleocapsid positivity and the number of prior anti-SARS-CoV-2 vaccine doses remained significantly associated with baseline anti-Spike titers. Non-reactive anti-nucleocapsid status was strongly associated with lower antibody levels (estimate −12, 197 U/mL, 95% CI: −15, 974 to −8, 420; p < 0.0001) compared reactive status, and higher number of prior doses was positively associated with titers (estimate 2, 397, 95% CI: 294 to 4, 499; p = 0.026). Full model results are presented in **Supplementary Table 20**.

**Table 4:** Multivariable analysis of factors associated with baseline anti-spike antibody titers.

Multivariable regression model with backward variable selection				
Parameter	Change in baseline anti-Spike antibody titers*			p-value
	Estimate	95% CI		
Anti-Nucleocapsid status at baseline				
Reactive	Ref	Ref	Ref	Ref
Non-reactive	-12, 197	-15, 974	-8, 420	<0.0001
N of vaccine prior doses				
Continuous	2, 397	294	4, 499	0.026

*Estimated by robust regression: MM-method. The factors cohort (A.1-2, A.3, B, C), sex (female, male), cancer status at enrolment (non-metastatic, metastatic), and timing of last dose (<6 months, 6–9 months, >9 months) did not add statistical significant information to the above model.

Anti-Nucleocapsid positivity was defined as a value ≥ 0.80 U/mL, measured using the Elecsys^®^ Anti-SARS-CoV-2 immunoassay (Roche Diagnostics). CI, confidence interval; p-value derived from the Wald Chi-Square test; Ref, Reference category.

### Cellular immune responses

Twenty-six patients were evaluable for the sub-study, of which three only had baseline blood samples, precluding their inclusion in follow-up comparisons. Additionally, patients from cohort B (n=4) were excluded because of their lymphoma or chronic lymphocytic leukemia (CLL) diagnosis, which is known to alter immune cell subpopulations (14, 15). As a result, the final analysis was performed on 19 patients (**Supplementary Table 21**), evaluating changes in circulating immune cells subpopulations following mRNA COVID-19 vaccination of patients with cancer.

**Figure 3:**
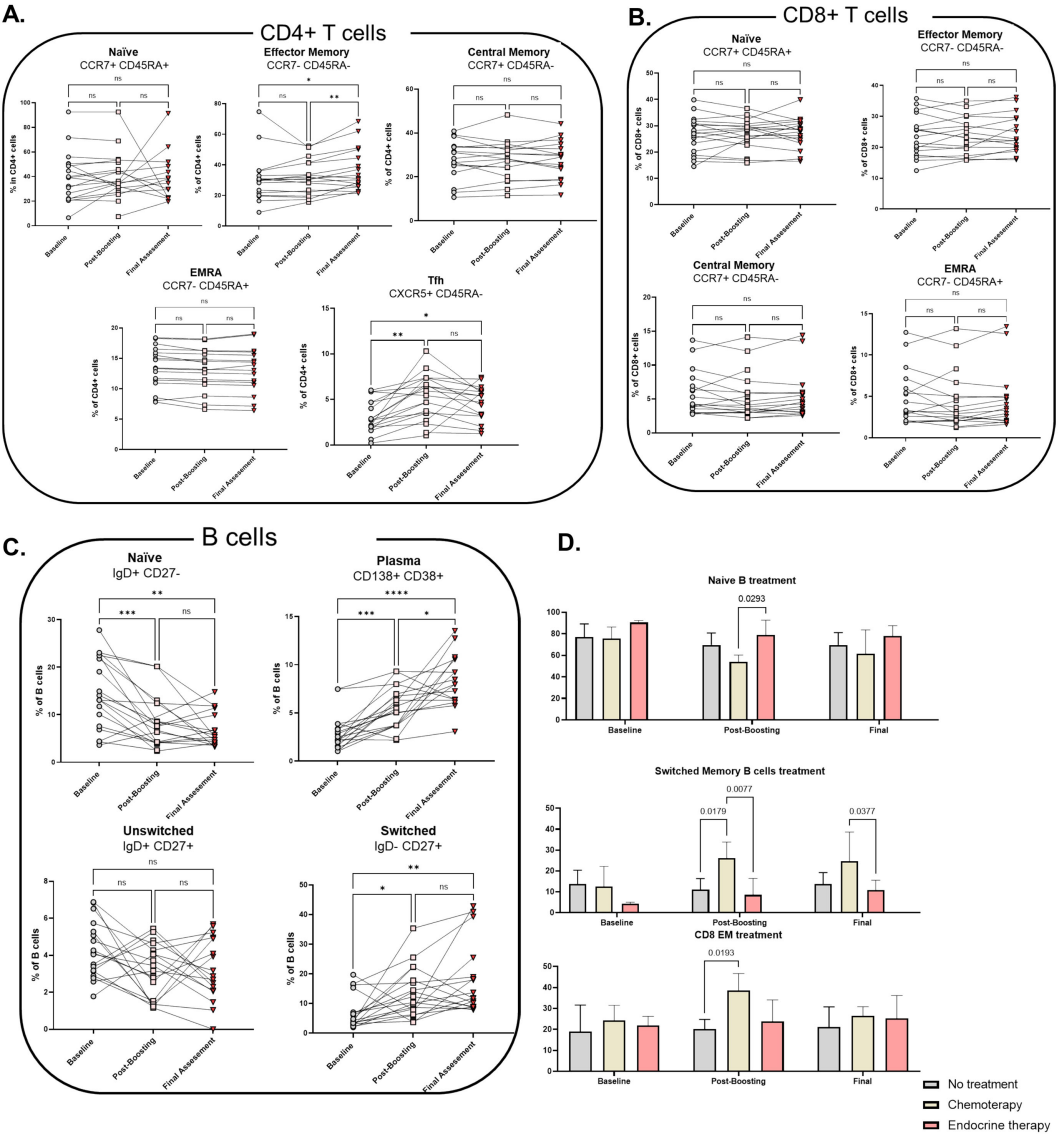
Longitudinal analysis of T and B cell subsets following SARS-CoV-2 mRNA vaccination in cancer patients. **(A)** CD4+ T cell subsets: naïve (CCR7+ CD45RA+), effector memory (CCR7− CD45RA−), central memory (CCR7+ CD45RA−), Tfh (CXCR5+ CD45RA−), and EMRA (CCR7− CD45RA+) at baseline, post-boost, and final assessment. **(B)** CD8+ T cell subsets: naïve, effector memory, central memory, and EMRA at baseline, post-boost, and final assessment. **(C)** B cell subsets: naïve (IgD+ CD27−), unswitched memory (IgD+ CD27+), switched memory (IgD− CD27+), and plasma cells (CD138+ CD38+). **(D)** Comparison of naïve B cells, switched memory B cells, and effector memory CD8+ T cells between patients in remission and those with active disease. Statistical analyses were performed using Tukey’s multiple comparisons test.

Significant alterations were observed in T cell and B cell subpopulations between baseline and post-boost assessments. Effector memory (EM; CCR7^-^CD45RA^-^) CD4^+^ T cells and T follicular helper (Tfh) cells (CXCR5^+^CD45RA^-^) were significantly increased at the post-boost timepoint, while other CD4^+^ subpopulations remained stable (**Figure 3A**). CD8^+^ T-cell subpopulations did not show significant changes following the booster vaccination (**Figure 3B**). Among B-cell subpopulations, the proportion of naïve B cells decreased between the baseline and post-boost samples, while the proportion of plasma cells (CD38^+^CD138^+^) and switched memory B cells (IgD^-^CD27^+^) increased (**Figure 3C**).

Further subgroup analyses revealed differences between patients receiving systemic treatment with those in remission (**Figure 3D**). In the absence of significant within-group longitudinal changes, we focused on cross-sectional comparisons between treatment groups at each timepoint. At post-boost, patients receiving chemotherapy had a lower proportion of naïve B cells compared to patients treated with endocrine therapy. Additionally, chemotherapy-treated patients had a higher proportion of switched memory B cells compared to untreated patients or those receiving endocrine therapy at both post-boost and final assessment. A higher proportion of CD8^+^ effector memory cells was also detected in chemotherapy-treated patients compared with untreated patients at the post-boost timepoint.

### SARS-CoV-2 infections

Among the 107 evaluable patients at baseline, 16% (n=17; 95% CI, 10–24%) went on to develop a confirmed SARS-CoV-2 infection during the study period (**Table 5**). Severe COVID-19 occurred in only 2 patients (2%; 95% CI, 0–7%) while 14 experienced symptomatic infections (13%; 95% CI, 7–21%) with only 1 (1%; 95% CI, 0–5%) having an asymptomatic infection.

**Table 5:** SARS-CoV-2 infection development.

SARS-CoV-2 infection development since last vaccination before ICF						
	Overall	Cohorts				p-value
		A.1–2	A.3	B	C	
Evaluable patients for baseline, N	107	56	17	9	25	-
SARS-CoV-2 Infection, n	17	9	1	2	5	0.602^b^
Rate of SARS-CoV-2 infection, % (95% CI)	16 (10-24)	16 (8-29)	6 (0-29)	22 (3-60)	20 (7-41)	-
Asymptomatic	1 (0-5)	2 (0-10)	0	0	0	–
Symptomatic	13 (7-21)	13 (5-24)	6 (0-29)	11 (0-48)	20 (7-41)	–
Severe	2 (0-7)	2 (0-10)	0	11 (0-48)	0	–
Seroconversion during follow-up, in those anti-Nucleocapsid non-reactive at baseline						
	Total	Cohorts				p-value
		A.1–2	A.3	B	C	
	N=37^a^	N=13	N=6	N=7	N=11	
Nucleocapsid becoming reactive during follow-up, n (%)						
No	26 (70)	11 (85)	5 (83)	5 (71)	5 (45)	0.211^b^
Yes	11 (30)	2 (15)	1 (17)	2 (29)	6 (55)	

Statistical test applied: (b) Fisher Exact test. (a) Only applicable for patients with nucleocapsid non-reactive at baseline and >1 blood sample during trial. Anti-Nucleocapsid positivity was defined as a value ≥ 0.80 U/mL, measured using the Elecsys^®^ Anti-SARS-CoV-2 N immunoassay (Roche Diagnostics). 95% CI, 95% confidence interval; ICF, informed consent signature; N, number; SARS-CoV-2, Severe Acute Respiratory Syndrome Coronavirus 2.

The incidence of SARS-CoV-2 infection varied across the cohorts: in cohort A.1-2, 9 of 56 patients (16%; 95% CI, 8–28%), in cohort A.3, 1 of 17 (6%; 95% CI, 0–29%), in cohort B, 2 of 9 (22%; 95% CI, 3–60%) and in cohort C, 5 of 25 (20%; 95% CI, 7–41%). A statistically significant association was not observed between cohort allocation and SARS-CoV-2 incidence (p=0.602).

Exploration of potential asymptomatic infections was accomplished by analyzing patients who were anti-Nucleocapsid negative at baseline and had at least one subsequent sample taken (n=37). Among these, 30% (n=11) seroconverted to anti-Nucleocapsid positivity during follow-up, consistent with a new SARS-CoV-2 exposure (**Table 5**). The seroconversion rate varied across cohorts, ranging from 15% in cohort A.1–2 to 55% in cohort C (p=0.211).

### Safety

No adverse events were reported during the trial. All patients tolerated the booster vaccines administered during the study without any related adverse events, serious adverse reactions or safety concerns at any time. No adverse reactions related to study procedures were documented.

## Discussion

This single-arm, phase IV, investigator-initiated clinical trial enrolled patients with a cancer diagnosis who were either receiving active systemic treatment or in remission without therapy for at least one year. The study centrally evaluated both humoral responses and balances of T and B cell subpopulations following administration of at least two doses of an anti-SARS-CoV-2 mRNA vaccine. Several key findings emerged, which have potentially important implications for optimizing vaccination strategies in this at-risk population.

One hundred percent seropositivity was observed across all cohorts both at baseline and subsequent timepoints, irrespective of cancer type or systemic therapy using the manufacturer-validated cut-off of the Elecsys^®^ Anti-SARS-CoV-2 S assay. This contrasts with the conclusions from our prior meta-analysis of over 30, 000 patients with cancer, which reported an overall seroconversion rate of 80% following complete primary vaccination (8). Importantly, seropositivity definitions varied across studies included in the abovementioned meta-analysis, reflecting the use of different immunoassays and assay-specific cut-offs, whereas the present study applied a single, standardized assay and its respective validated threshold. These I-SPARC data reinforce the robust immunogenicity of mRNA vaccines, even in immunocompromised populations and expand prior prospective studies, such as the VOICE trial (12), via the extended follow-up, detailed immunophenotyping and inclusion of patient populations that are often underrepresented in vaccination research. To contextualize these findings, published studies in immunocompetent populations using standardized anti-Spike antibody assays, have reported a wide range of antibody levels depending on timing and vaccine exposure, typically ranging from approximately 1,000 to 8,000 BAU/mL following primary mRNA vaccination series, and exceeding 20,000 BAU/mL after booster doses (16). In this context, the absolute antibody levels observed in cohort A.1–2 of our study fall within the range reported in healthy populations. However, validity of direct cross-study comparisons remains limited by several factors including potential differences in sampling timepoints, prior SARS-CoV-2 infection, and cumulative vaccine exposure.

Among the patients with solid tumors, chemotherapy administration (cohort A.3) was associated with significantly lower anti-Spike antibody titers compared to those receiving other systemic therapies. This observation is consistent with earlier reports documenting chemotherapy-induced immunosuppression (9, 17, 18). Previous studies have reported seroconversion rates as low as 45% in patients receiving chemotherapy; however, administration of a third dose significantly improved response rates, supporting the role of recurrent antigenic stimulation in overcoming treatment-related immune deficits (19). In I-SPARC, booster doses similarly increased antibody titers in chemotherapy-treated patients, reinforcing their utility in enhancing humoral immune responses (20). Anti-Spike antibody levels increased following booster vaccination and declined at the final assessment, with substantial inter-individual variability in the magnitude of waning. Despite these increases, antibody levels in chemotherapy recipients remained lower than in the non-chemotherapy cohorts, underscoring the need for tailored vaccination strategies, including optimized booster schedules and potentially adjunctive immunomodulatory interventions (20). However, chemotherapy exposure was not independently associated with anti-Spike antibody titers in the multivariable analysis, suggesting that the lower antibody levels observed in univariable analyses are at least partly mediated by other clinical factors, such as prior SARS-CoV-2 infection and cumulative vaccination.

Although seroconversion was achieved in all participants, patients with hematologic malignancies exhibited significantly lower absolute anti-Spike antibody titers compared to those with solid tumors, in line with prior evidence (18, 21). In our previous meta-analysis, patients with solid tumors had significantly higher seroconversion rates (94%) compared to those with hematologic malignancies (60%; odds ratio [OR], 0.35; 95% CI, 0.18–0.69; p = 0.002) (8). Additional studies, including CAPTURE and SerOzNET, reported seroconversion rates ranging from 59% to 85% in hematologic malignancies following two to three vaccination doses – rates consistently lower than in solid tumors (18, 21). The majority of patients in cohort B had B-cell malignancies, which are known to impair vaccine responses due to both disease-related and treatment-related immunosuppression (9, 22, 23). Patients receiving anti-CD20 therapy had markedly reduced antibody titers, as also shown in LymphoCOVAC and Šušol’s studies, highlighting the importance of vaccination timing in relation to B-cell reconstitution (9, 22, 23). The SerOzNET study further showed that patients with hematologic malignancies often required supplementary vaccinations, with some responding only after a fourth or fifth dose (21). These findings collectively underscore the importance of monitoring both humoral and cellular immune responses in this population and identifying patients that may benefit from intensified protection strategies, including prophylactic monoclonal antibodies. However, the heterogeneity of hematologic diseases and treatments within this cohort should be considered when interpreting these findings, as it may limit the generalizability of results. Although anti-Spike antibody titers are widely used as a surrogate marker of humoral immunity, their correlation with clinical protection, particularly against immune-evasive variants such as Omicron, remains imperfect and should be interpreted with caution. In addition, the absence of neutralizing antibody assays limits the ability to directly assess functional antibody responses.

A key strength of the I-SPARC trial is the inclusion of patients with disease remission (cohort C), featuring patients with a prior history of cancer diagnosis but without systemic therapy for at least one year with no evidence of disease. This population is frequently excluded from immunogenicity studies despite representing a growing segment of cancer survivors. Patients in this cohort had consistently high and sustained antibody titers that were comparable to those detected in patients receiving non-cytotoxic systemic therapies. These findings suggest that immune competence is preserved or has recovered in remission and highlight the importance of including this subgroup in future vaccine research and health policy strategies.

Prior SARS-CoV-2 infection, centrally determined by anti-Nucleocapsid antibody positivity, was associated with significantly higher anti-Spike antibody titers at all timepoints, supporting the concept of hybrid immunity – where prior antigen exposure enhances vaccine-induced humoral responses (12, 18, 19). This is consistent with studies showing that individuals with prior SARS-CoV-2 infection generate more robust immune responses post-vaccination, including improved neutralizing antibody levels and enhanced T-cell responses (17, 24). Furthermore, hybrid immunity has been linked in previous reports to a reduced risk of hospitalization (OR, 0.39; p=0.01) and a lower likelihood of breakthrough infections, particularly in patients with hematologic malignancies, who often have suboptimal vaccine responses (19, 20, 25, 26). These findings underscore the importance of prior antigen exposure for boosting vaccine efficacy and support ongoing immune monitoring in patients with cancer.

Subgroup analyses also provided insight into the heterogeneity of vaccine responses based on demographic and clinical factors. Contrary to earlier reports suggesting an age-related decline in vaccine immunogenicity (27, 28), I-SPARC did not detect a significant reduction in antibody titers among older patients. This aligns with findings by Kobayashi et al., who found that while patients aged ≥75 years initially showed lower antibody responses after two doses, subsequent booster vaccinations equalized titers between older and younger individuals (29). These data suggest that booster vaccinations play a critical role in mitigating the effects of immunological aging by achieving comparable endpoint immunity for all age groups.

Although differences between male and female patients did not contribute to predict the humoral immunity in our multivariable model, a trend toward lower absolute anti-Spike titers was detected in the male patients. This is consistent with previous studies reporting sex-based differences in humoral immunity, with females typically having stronger antibody responses following vaccination (10, 17–20, 30). Men have been shown to have a higher risk of severe COVID-19 outcomes, including hospitalization (19, 20, 25), further emphasizing the potential clinical relevance of these immunological differences.

In our study, BMI was also a factor influencing vaccination responses in the overall population. At the post-boost timepoint, overweight and obese individuals had significantly higher anti-Spike antibody titers compared to healthy-weight individuals. This observation supports previous studies linking an elevated BMI to heightened humoral responses following anti-SARS-CoV-2 vaccination (12, 25, 27). The immunological mechanisms underlying this association may reflect a baseline inflammatory state that favors vaccine-induced immune activation (27). The relevance of this phenomenon for patients with cancer remains unclear particularly because metabolic dysregulation and tumor-related immune effects could modulate their vaccination responsiveness.

Multivariable analysis identified prior SARS-CoV-2 infection and the total number of anti-SARS-CoV-2 vaccinations as the strongest independent predictors of higher anti-Spike antibody titers. This finding is aligned with previous studies that have shown repeated antigen exposure – through natural infection or additional vaccination – enhances vaccine-induced immunity (18, 19, 27). Additional vaccinations have been associated with a 2.4-fold increase in antibody titers, with the third and fourth doses improving responses across cancer types, albeit with persistently reduced responses in patients receiving B-cell-depleting therapies (12, 20). Data from the MELODY study by Mumford et al. indicated that cumulative vaccination is associated with a reduced risk of hospitalization, further underscoring the clinical utility of booster strategies (25). Our results highlight the need for personalized booster strategies, particularly for patients with suboptimal responses to primary vaccination.

During the I-SPARC study, 16% of evaluable patients developed a confirmed SARS-CoV-2 infection. This frequency, during widespread community transmission driven by the Omicron variants, is substantially lower than the 29% infection rate reported in other prospective cancer cohorts, such as the SerOzNET study (21). Only two severe cases occurred in I-SPARC with no reported COVID-19-related deaths, supporting the protective role of mRNA vaccination, particularly in combination with booster vaccines and sustained T-cell immunity, in reducing the risk of severe disease among immunocompromised individuals.

The I-SPARC trial provides some mechanistic insights into immune responses to mRNA vaccination in cancer patients. We observed distinct alterations in T- and B-cell subpopulations, including an increase in effector memory CD4^+^ T cells and Tfh cells, which provide long-term immunological memory and essential help for antibody production, respectively. CD8^+^ T-cell subpopulations were more stable, which suggests that cytotoxic T-cell responses are less affected or responsive to vaccination in these patients. The decline in naïve B cells and corresponding increase in plasma cells plus switched memory B cells are characteristic of active B-cell differentiation and maturation following vaccination. These findings are consistent with previous studies demonstrating that cancer patients, including those with diminished neutralizing antibody responses, can mount functional T-cell responses post-vaccination (12, 18, 22). T-cell immunity is known to be particularly important for protection against severe disease (19, 20).

The subgroup analyses revealed that, compared with patients receiving endocrine therapy or no systemic treatment, those receiving chemotherapy had lower levels of naïve B cells, and higher proportions of both switched memory B cells and CD8^+^ effector memory T cells. These findings indicate treatment-associated differences in post-vaccination immune cell subset distributions, which is in agreement with previous reports describing chemotherapy-induced changes in B-cell differentiation (17, 21, 31). The elevated CD8^+^ effector memory responses observed in chemotherapy-treated patients could reflect compensatory mechanisms in the context of reduced humoral immunity (25). Patients with lymphoma or CLL were excluded from our analysis due to their known immunological deficiencies based on previous studies confirming that B-cell-depleting therapies, such as anti-CD20 monoclonal antibodies, impair both their T- and B-cell responses (22, 23).

However, the I-SPARC study is not without limitations. First, the absence of an age-matched healthy control group limits direct quantitative comparison of absolute anti-Spike antibody titers with the general population. Second, the rapidly evolving nature of the SARS-CoV-2 pandemic necessitated multiple amendments to the study protocol, which impacted trial execution and introduced variability in data collection. Third, the original design expected to enroll 400 evaluable patients (100 per cohort), but slow recruitment led to early trial closure before this target was achieved. This limited the statistical power for subgroup analyses, which should therefore be considered exploratory and hypothesis-generating. In addition, the number of subgroup and pairwise comparisons increases the risk of chance findings, and results should be interpreted primarily by magnitude and consistency rather than statistical significance alone. Moreover, the low number of severe COVID-19 cases observed (n=2) precluded any formal assessment of the relationship between antibody titers and the risk of severe SARS-CoV-2 infection among the patients included in our study. Additionally, the study was not activated in Portugal, further restricting accrual to Belgium only. Finally, although quantification of antibody titers serves as a useful surrogate for humoral immunity, it does not fully reflect functional neutralizing activity or cellular immune competence, both of which require complementary assessments.

Beyond its immediate clinical and health policy implications, the I-SPARC trial contributes to the growing body of literature supporting the immunogenicity of mRNA vaccine platforms in patients with cancer. The success of mRNA platforms against SARS-CoV-2 has accelerated their translation into oncologic applications, underscoring the importance of coordinated humoral and cellular immune responses in immunocompromised populations (32, 33). In this context, our findings support the immunogenic potential of mRNA platforms for cancer patients and these data provide translational insights that may inform the rational development and evolution of future mRNA-based cancer vaccines (32, 34).

Our results have direct implications for clinical practice and health policy strategies to manage patients with cancer. Patients receiving or scheduled to receive chemotherapy should be prioritized for timely mRNA vaccination to overcome the lower antibody titers during chemotherapy and thereby derive benefit from pre-treatment immunization (22). Booster vaccination remains a critical component of achieving durable responses in immunocompromised populations, which is consistent with previous studies (23, 35). As mRNA vaccine technologies evolve, key challenges remain, including optimizing tumor antigen selection, improving mRNA stability and enhancing vaccine delivery (36, 37). Innovations such as AI-driven neoantigen prediction and machine learning–based immune profiling may help to personalize vaccine design and improve patient selection (38).

In summary, the results from the I-SPARC trial provide prospectively acquired evidence that supports the robust immunogenicity of SARS-CoV-2 mRNA vaccines in patients with cancer, including those with weakened immune responses. Enhancement of humoral responses with booster vaccination, particularly for patients with weakened immunity, as well as previous infections bestowed additional benefit through hybrid immunity. Preserved T-cell responses across the study population further emphasize the complementary role of cellular immunity in vaccine-induced protection. Our findings support a personalized approach to vaccination in oncology, in which immune status, prior exposure and treatment history should be considered when planning vaccination strategies, including the potential need for boosters or passive immunoprophylaxis.

## Methods

### Study design

**Figure 4:**
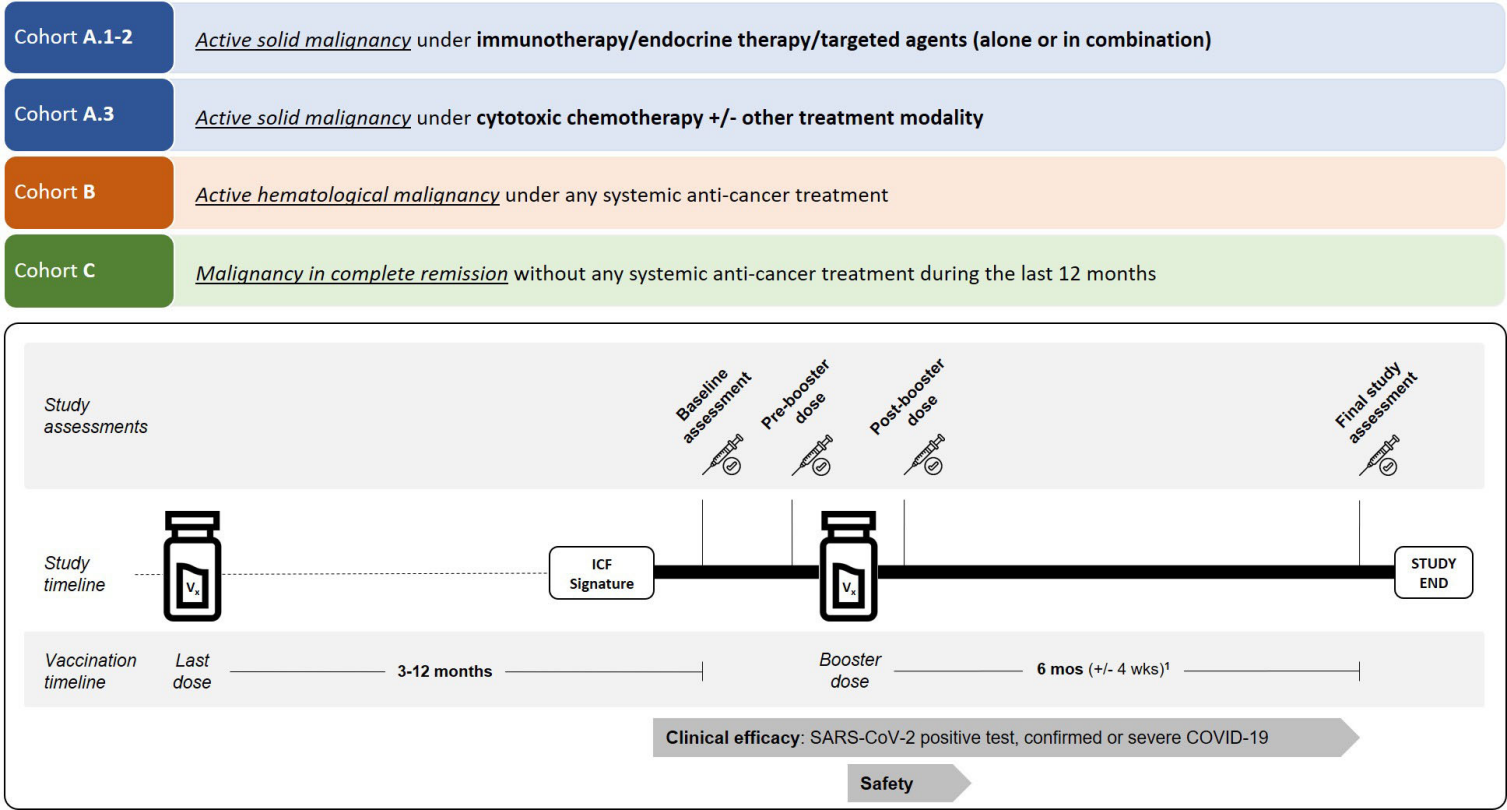
Study design and assessment timeline of the I-SPARC trial. Study design of the I-SPARC trial. The study consists of four cohorts (A1-2, A3, B, C). Blood samples for immune response assessment were collected at predefined time points: baseline (3–12 months after the last vaccine dose administered prior to study enrolment), pre-booster (within 2 weeks before a booster dose, if the booster was not administered within two weeks of the baseline assessment), post-boost (2 weeks ±3 days after a booster dose), and final study assessment (6 months ±4 weeks after baseline assessment or 6 months ±4 weeks after a booster dose if administered during the study). If a booster dose was scheduled before 6 months minus 4 weeks after the last dose, the final study assessment could be conducted up to 6 months minus 8 weeks. Blood samples were collected as close as possible to the scheduled 6-month time points to ensure data consistency. All patients were monitored throughout the study for clinical efficacy, including asymptomatic SARS-CoV-2 positivity and confirmed or severe COVID-19. Safety assessments were conducted for 30 days following any booster dose, focusing on potential adverse reactions.

The I-SPARC study (NCT05075538; EudraCT 2021-003710-39) was an investigator-initiated, multicenter, open-label, single-arm, non-randomized phase IV clinical trial. The primary objective was to evaluate the long-term immune response to mRNA-based anti-SARS-CoV-2 vaccination in patients with a cancer diagnosis. The study design and procedures are summarized in **Figure 4**. Given the rapidly evolving landscape of the COVID-19 pandemic and associated vaccine strategies, the protocol underwent multiple amendments, with Version 6.0 serving as the final approved version (**Annex II**). This manuscript was prepared in accordance with the Transparent Reporting of Evaluations with Nonrandomized Designs (TREND) statement (39), ensuring comprehensive and transparent reporting of this non-randomized interventional study.

### Participants

Patients were identified through routine oncology care and recruited predominantly in the outpatient setting, including during scheduled clinic visits or treatment appointments. Eligible participants were adult men and women with a diagnosis of invasive solid tumors or hematologic malignancies who were either receiving systemic therapy or in complete remission without active treatment for at least 12 months. Eligibility required receipt of at least two doses of an mRNA-based SARS-CoV-2 vaccine – either Comirnaty^®^ (BNT162b2, Pfizer/BioNTech) or Spikevax^®^ (mRNA-1273, Moderna) – in accordance with national vaccination guidelines. Initially, inclusion was restricted to patients who had received exactly two vaccine doses within six months prior to enrollment. However, following a protocol amendment in Jul 2022, eligibility was expanded from allowing only patients with exactly two doses of mRNA vaccine within six months to including those with at least two doses, and a protocol amendment in Feb 2023 with the last dose had been administered between 3 and 12 months before the baseline visit (detailed eligibility criteria in **Supplementary Table 22**). Patients were allocated into predefined cohorts based on type of cancer and treatment status at the time of their last vaccination dose before informed consent form (ICF) signature. Patients with active solid malignancies were assigned to cohort A.1–2 if receiving immunotherapy, targeted agents, or endocrine therapy without cytotoxic chemotherapy, or cohort A.3 if undergoing cytotoxic chemotherapy, either alone or in combination with other treatments. Patients with active hematologic malignancies receiving systemic treatment were allocated to cohort B, while patients in complete remission without systemic cancer treatment for at least one year were assigned to cohort C, irrespective of type of cancer. Eligibility was determined at enrolment, whereas cohort assignment and sample evaluability were assessed at each study timepoint. Patients were considered evaluable only if they met the cohort-defining criteria at the time of at the relevant vaccination-associated assessment (e.g., receiving active systemic therapy or in remission without systemic treatment for ≥12 months). Patients with active cancer without ongoing systemic therapy, as well as patients in remission for less than 12 months since their last systemic treatment, were not considered eligible at screening or evaluable at that specific timepoint but could contribute evaluable samples at subsequent visits if criteria were fulfilled (**Supplementary Figure 1**; **Annex I**).

Originally, five cohorts were planned; however, based on emerging evidence suggesting similar short-term immune responses to primary vaccination among patients receiving immunotherapy, targeted agents, or endocrine therapy (9), cohorts A.1 (immunotherapy) and A.2 (targeted/endocrine therapy) were merged into a single cohort A.1–2, which included patients receiving immunotherapy, targeted agents, or endocrine therapy, alone or in combination (excluding cytotoxic chemotherapy).

### Objectives and endpoints

The primary objective of the I-SPARC trial was to evaluate the long-term humoral immune response against SARS-CoV-2 in patients with cancer diagnosis, assessed between 3 and 12 months after the last dose of a mRNA anti-SARS-CoV-2 vaccine administered prior to study enrolment (baseline assessment), measured by the seropositive rate for anti-Spike protein antibodies, defined according to the manufacturer-validated cut-off of the Elecsys^®^ Anti-SARS-CoV-2 S assay (Roche Diagnostics; ≥0.8 U/mL).

The secondary objectives were to: (1) assess the durability of the humoral immune response at the final study assessment, based on the proportion of patients with detectable anti-Spike protein antibodies at that timepoint; (2) determine the clinical efficacy of SARS-CoV-2 vaccination, evaluated by the proportion of patients who developed asymptomatic SARS-CoV-2 infection, confirmed COVID-19, or severe COVID-19; and (3) evaluate the safety of booster doses administered during the study, assessed by the frequency, duration, and severity of adverse reactions, classified according to NCI Common Terminology Criteria for Adverse Events v5.0.

In addition to these predefined objectives, *post hoc* analyses were conducted to further investigate dynamics of humoral immune response following anti-SARS-CoV-2 vaccination. These analyses provided a more detailed characterization of absolute anti-Spike antibody titers across cohorts and their variations over time. The impact of prior SARS-CoV-2 infection (anti-Nucleocapsid positivity, centrally tested with Elecsys^®^ Anti-SARS-CoV-2 N assay), along with other subgroup analyses, was explored to assess potential differences in anti-Spike antibody titers based on demographic and clinical factors.

### Study procedures and assessments

The study procedures and assessment timeline are illustrated in **Figure 4**. Blood samples for immune response assessment were collected at predefined time points: baseline (3–12 months after the last vaccine dose administered prior to study enrolment), pre-booster (within 2 weeks before a booster dose, if the booster was not administered within two weeks of the baseline assessment), post-boost (2 weeks ±3 days after a booster dose), and final study assessment (6 months ±4 weeks after baseline assessment or 6 months ±4 weeks after a booster dose if administered during the study). Demographics and medical history, anti-cancer treatments, anti-SARS-CoV-2 vaccination records, and routine laboratory assessments, and diagnosis of COVID-19 were assessed at screening and at each study assessment. Peripheral serum blood samples were collected for humoral immune response assessment at the above-specified timepoints, and adverse events related to these procedures and the booster doses received on study were collected throughout the study period. A pre-defined subset of up to 20 patients selected from the main study population at Institut Jules Bordet were consented to participate in a sub-study evaluating cellular immune response, from whom additional whole blood samples were collected at the same timepoints.

#### Immune response evaluation

The humoral immune response was assessed using semi-quantitative and qualitative immunoassays. Peripheral serum blood samples were collected, stored at -80 °C before centralized testing, and analyzed centrally in batches to ensure consistency. The Elecsys^®^ Anti-SARS-CoV-2 S assay (Roche Diagnostics, Vilvoorde, Belgium) was used to detect total anti-Spike antibodies, which may result from both vaccination and natural infection. Results are reported in U/mL; according to the manufacturer, assay units are traceable to the WHO international standard, with 1 U/mL corresponding to 1 binding antibody unit (BAU/mL). This assay was centrally performed on a Cobas e801 analyzer, with results interpreted as follows: <0.8 U/mL (negative) or ≥0.8 U/mL (positive). For samples with values >250 U/mL, dilution was performed to determine the exact antibody titer.

In addition, the Elecsys^®^ Anti-SARS-CoV-2 N assay (Roche Diagnostics, Vilvoorde, Belgium) was used to detect total anti-Nucleocapsid antibodies, which are exclusively indicative of natural infection. This test was also centrally conducted on a Cobas e801 analyzer, with results reported as negative (cut-off index [COI] <1) or positive (COI ≥1) for anti-SARS-CoV-2 antibodies.

For the cellular immune response in the patients participating to the sub-study, peripheral blood mononuclear cells (PBMCs) were isolated from the whole blood sample and washed before further processing. A total of 5 x 10^5^ PBMCs were incubated with fluorescently labeled primary monoclonal antibodies, diluted according to the manufacturer’s recommendations (**Supplementary Table 23**), for 30 minutes at 4 °C. The cells were then washed with phosphate-buffered saline (PBS) and resuspended in a red blood cell lysis solution, followed by a 10-minute incubation at room temperature. After a final wash, PBMCs were acquired using a GALLIOS 10/3 flow cytometer, and data analysis was performed with Kaluza Flow Cytometry Analysis v1.2 software (Beckman Coulter). Standardized protocols were applied for cell isolation, antibody staining, and data analysis to ensure consistency and reproducibility.

All humoral and cellular immune response assessments were performed centrally at the Institut Jules Bordet central and immunology laboratory, respectively, using standardized protocols.

### Statistical analyses

The trial was planned to recruit 440 participants, assuming a drop-out rate of 10% to obtain 400 evaluable participants, 100 for each cohort in order to estimate the rate of immune response in a cohort with a 95% interval of maximum width of 20%. Due to slow recruitment, the study was terminated early before reaching the planned enrollment. As a result, the study was underpowered for formal comparisons across certain subgroups, particularly for cohort B. Evaluability in the I-SPARC trial was defined as patients with available blood samples at the predefined study timepoints and with histologically or cytologically confirmed cancer diagnosis undergoing active systemic cancer treatment for non-metastatic/curative or metastatic/palliative setting, or those undergoing follow-up after confirmed cancer complete remission without active cancer treatment for the last 12 months, at the time of the last dose of the mRNA anti-SARS-CoV-2 vaccination before study entry or at booster vaccination on study period. Details of patients evaluable and excluded from the analysis are provided in **Supplementary Figure 1**; **Annex I**. Descriptive statistics were used to summarize baseline characteristics, with median and interquartile ranges (IQR) for continuous variables and frequencies and percentages for categorical variables. The Kruskal-Wallis test was used to compare continuous variables across cohorts, and the Fisher’s exact test was applied for categorical variables. If an overall p-value <0.10 was observed, pairwise comparisons between cohorts were performed without adjustment for multiple testing. Given the number of comparisons, the limited sample size, due to early termination of the study, resulting in small subgroups, these subgroup analyses should be interpreted as exploratory and hypothesis-generating.

Analyses of absolute anti-Spike antibody titers were conducted to compare values across cohorts at the predefined study timepoints, and additional stratified subgroup analyses (e.g. anti-Nucleocapsid positivity [reactive vs. non-reactive], age, sex, etc.).

The longitudinal evolution of antibody titers over time was analyzed within individual patients, with cohort classification based on treatment status at the time of measurement. Statistical comparisons were not performed for subgroups with fewer than five observations per timepoint, in line with good statistical practice and to avoid unstable estimates, increased risk of Type I and Type II errors, and unreliable inference. Multivariable analysis was performed using a robust regression based on the MM method to examine associations between baseline anti-Spike antibody titers and clinical factors, adjusting for clinically relevant and/or statistically significant variables. All statistical analyses were performed using SAS (version 9.4).

Statistical analyses for the flow cytometry were performed with GraphPad Prism software (version 10). Data are represented as individual values. Statistical significance was determined using a Tukey’s multiple comparison test.

## Data Availability

The data that support the conclusions of this study are included in the article, and Supplementary Data file.
