# Supplementary material for: Long-term immune response to mRNA anti-SARS-CoV-2 vaccination in patients with cancer
Source: Front Immunol. 2026 May 20;17:1815933. doi: 10.3389/fimmu.2026.1815933 (PMC13231279; doi:10.3389/fimmu.2026.1815933)
Supplement: Supplementary file 1 [file DataSheet1.docx]

**Supplementary data**

Supplementary Figures

[Supp. Fig. S 1. Patient Enrollment and Eligibility Flowchart in the I-SPARC Trial. 2](#_Toc191462969)

[Supp. Fig. S 2. Cohort Transitions and Booster Vaccination Status in the I-SPARC Trial. 3](#_Toc191462970)

[Supp. Fig. S 3. Longitudinal Anti-Spike Titers in Cohort A.1-2. 4](#_Toc191462971)

[Supp. Fig. S 4. Longitudinal Anti-Spike Titers in Cohort A.3. 5](#_Toc191462972)

[Supp. Fig. S 5. Longitudinal Anti-Spike Titers in Cohort B. 6](#_Toc191462973)

[Supp. Fig. S 6. Longitudinal Anti-Spike Titers in Cohort C. 7](#_Toc191462974)

Supplementary Tables

[Table S 1. Characteristics of patients in cohort B (hematological cancers under active treatment). 8](#_Toc220946538)

[Table S 2. Absolute titers levels of anti-SARS-CoV-2 spike at pre-booster assessment 9](#_Toc220946539)

[Table S 3. Test statistics of paired comparisons of anti-Spike antibody titers between study timepoints (Wilcoxon signed-rank). 10](#_Toc220946540)

[Table S 4. Anti-SARS-CoV-2 spike at baseline according to nucleocapsid status in the different cohorts 11](#_Toc220946541)

[Table S 5. Anti-SARS-CoV-2 spike at post-booster according to nucleocapsid status in the different cohorts 12](#_Toc220946542)

[Table S 6. Anti-SARS-CoV-2 spike at final assessment according to nucleocapsid status in the different cohorts. 13](#_Toc220946543)

[Table S 7. Subgroup analyses by age, anti-SARS-CoV-2 spike at baseline. 14](#_Toc220946544)

[Table S 8. Subgroup analyses by age, anti-SARS-CoV-2 spike at post-booster. 14](#_Toc220946545)

[Table S 9. Subgroup analyses by age, anti-SARS-CoV-2 spike at final assessment. 15](#_Toc220946546)

[Table S 10. Subgroup analyses by sex, anti-SARS-CoV-2 spike at baseline. 15](#_Toc220946547)

[Table S 11. Subgroup analyses by sex, anti-SARS-CoV-2 spike at post-booster. 16](#_Toc220946548)

[Table S 12. Subgroup analyses by sex, anti-SARS-CoV-2 spike at final assessment. 16](#_Toc220946549)

[Table S 13. Subgroup analyses by BMI classification, anti-SARS-CoV-2 spike at baseline. 17](#_Toc220946550)

[Table S 14. Subgroup analyses by BMI classification, anti-SARS-CoV-2 spike at post-booster. 17](#_Toc220946551)

[Table S 15. Subgroup analysis by age classification, anti-SARS-CoV-2 spike at pre-booster. 18](#_Toc220946552)

[Table S 16. Subgroup analysis by sex classification, anti-SARS-CoV-2 spike at pre-booster. 18](#_Toc220946553)

[Table S 17. Subgroup analysis by overweight/obesity, anti-SARS-CoV-2 spike at pre-booster. 19](#_Toc220946554)

[Table S 18. Subgroup analysis by smoker status, anti-SARS-CoV-2 spike at pre-booster. 19](#_Toc220946555)

[Table S 19. Subgroup analysis by absolute lymphocyte count at baseline, anti-SARS-CoV-2 spike at pre-booster. 20](#_Toc220946556)

[Table S 20. Multivariable robust regression model. 21](#_Toc220946557)

[Table S 21. Characteristics of patients included in the flow cytometry analysis. 22](#_Toc220946558)

[Table S 22. Eligibility criteria for the I-SPARC trial, protocol version 6.0. 23](#_Toc220946559)

[Table S 23. Antibodies used for flow cytometry analysis 24](#_Toc220946560)

**Supplementary Fig. S1.**

Supp. Fig. S 1. Patient Enrollment and Eligibility Flowchart in the I-SPARC Trial.

Flowchart depicting patient enrolment, eligibility assessment, and inclusion in the I-SPARC trial. Of the 152 patients assessed for eligibility, 11 were excluded due to predefined exclusion criteria, including active leukemia (n=1), receipt of non-mRNA SARS-CoV-2 vaccination (n=9), recent COVID-19 infection (n=1), or lack of available blood samples (n=1). The remaining 137 were assessed to check their eligibility regarding inclusion criteria (IC) 3: "Cancer diagnosis, with active systemic treatment or in remission without treatment for ≥12 months, at last vaccination before enrolment" (as per protocol version 6.0). Of these, 30 patients did not meet this eligibility criterion. However, some of these patients were considered evaluable because they received a booster vaccination during the study period and met IC3 at the time of the booster dose, with evaluable blood samples available for analysis. As a result, 115 patients were included in the final analysis. This flowchart provides a comprehensive overview of patient selection and cohort allocation throughout the study. Abbreviations: COVID-19, Coronavirus Disease 2019; IC3, Inclusion Criterion 3; mRNA, Messenger Ribonucleic Acid; SARS-CoV-2, Severe Acute Respiratory Syndrome Coronavirus 2.

**Supplementary Fig. S2.**

| 1. Patients in cohort A.1-2 at enrolment. | 1. Patients in cohort A.3 at enrolment. |
| --- | --- |
| 1. Patients in cohort B at enrolment. | 1. Patients in cohort C at enrolment. |

Supp. Fig. S 2. Cohort Transitions and Booster Vaccination Status in the I-SPARC Trial.

Flowcharts illustrating cohort allocation and transitions in the I-SPARC trial. The figure is divided into four panels: a, b, c, and d. Panel a shows patients in Cohort A.1-2 at enrollment, Panel b shows patients in Cohort A.3 at enrollment, Panel c shows patients in Cohort B at enrollment, and Panel d shows patients in Cohort C at enrollment. Each panel illustrates the initial cohort distribution based on the treatment status at the time of the last vaccination before informed consent (ICF) signature. Patients who received a booster vaccination on study were reassessed, and if their treatment status had changed, they were reassigned to the corresponding cohort at the time of booster vaccination. Patients who did not receive a booster remained in their initial cohort. Notes: cohort A.1-2, Active solid malignancies under immunotherapy, endocrine therapy, or targeted agents (excluding chemotherapy); cohort A.3, Active solid malignancies under cytotoxic chemotherapy; cohort B, Active hematologic malignancies under systemic treatment; cohort C, Patients in complete remission without systemic treatment for at least one year.

**Supplementary Fig. S3.**


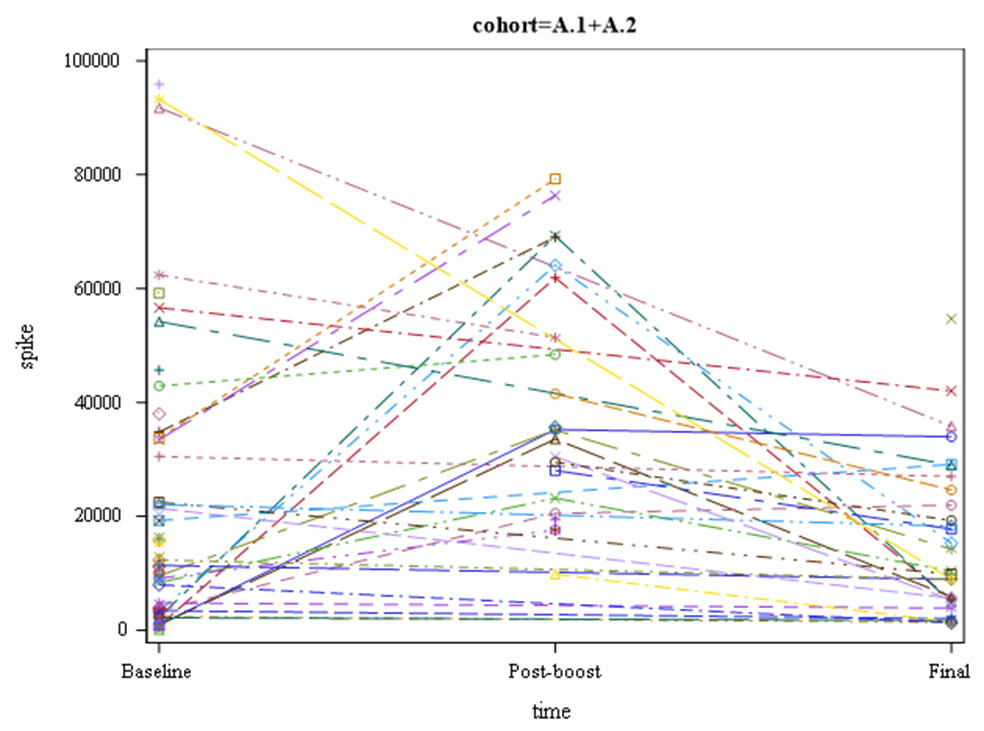


Supp. Fig. S 3. Longitudinal Anti-Spike Titers in Cohort A.1-2.

Individual anti-SARS-CoV-2 spike antibody trajectories in Cohort A.1-2 from baseline to post-booster and final assessment. Each line represents one patient, showing variation in antibody responses over time.

**Supplementary Fig. S4.**


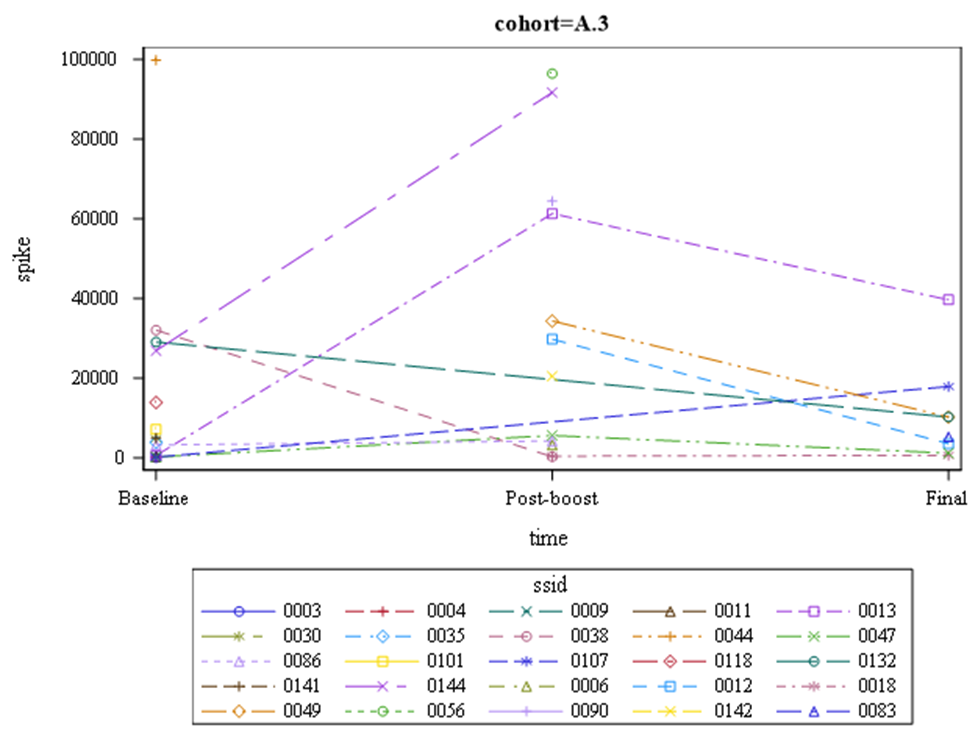


Supp. Fig. S 4. Longitudinal Anti-Spike Titers in Cohort A.3.

Individual anti-S antibody trajectories in Cohort A.3 from baseline to post-booster and final assessment. Each line represents one patient, showing variation in antibody responses over time.

**Supplementary Fig. S5.**


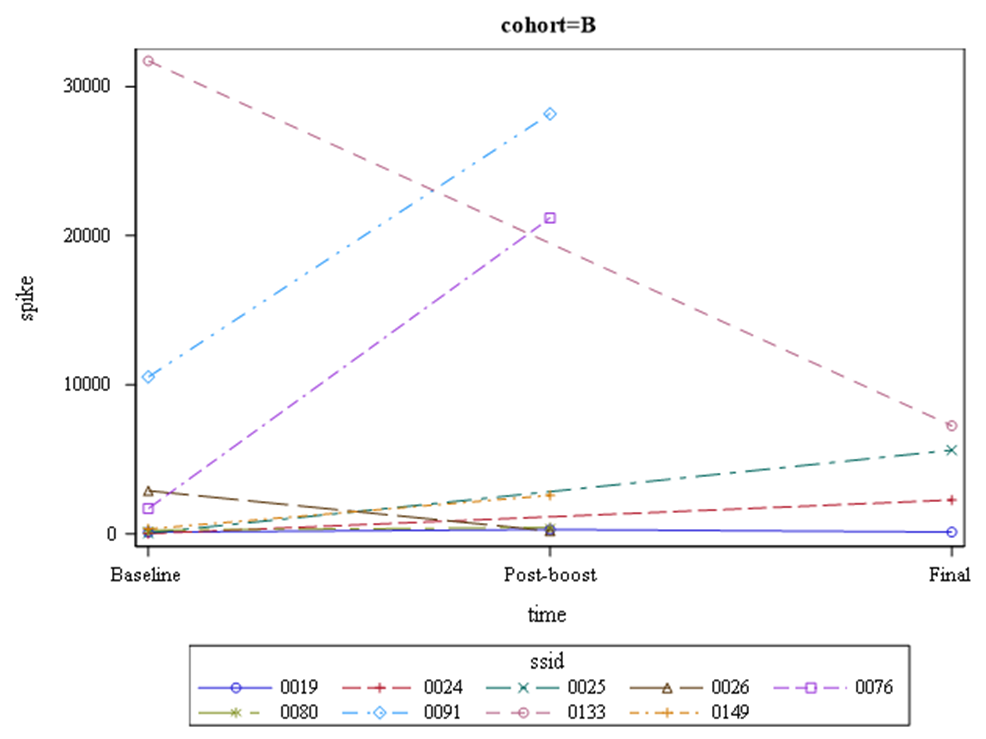


Supp. Fig. S 5. Longitudinal Anti-Spike Titers in Cohort B.

Individual anti-S antibody trajectories in Cohort B from baseline to post-booster and final assessment. Each line represents one patient, showing variation in antibody responses over time.

**Supplementary Fig. S6.**


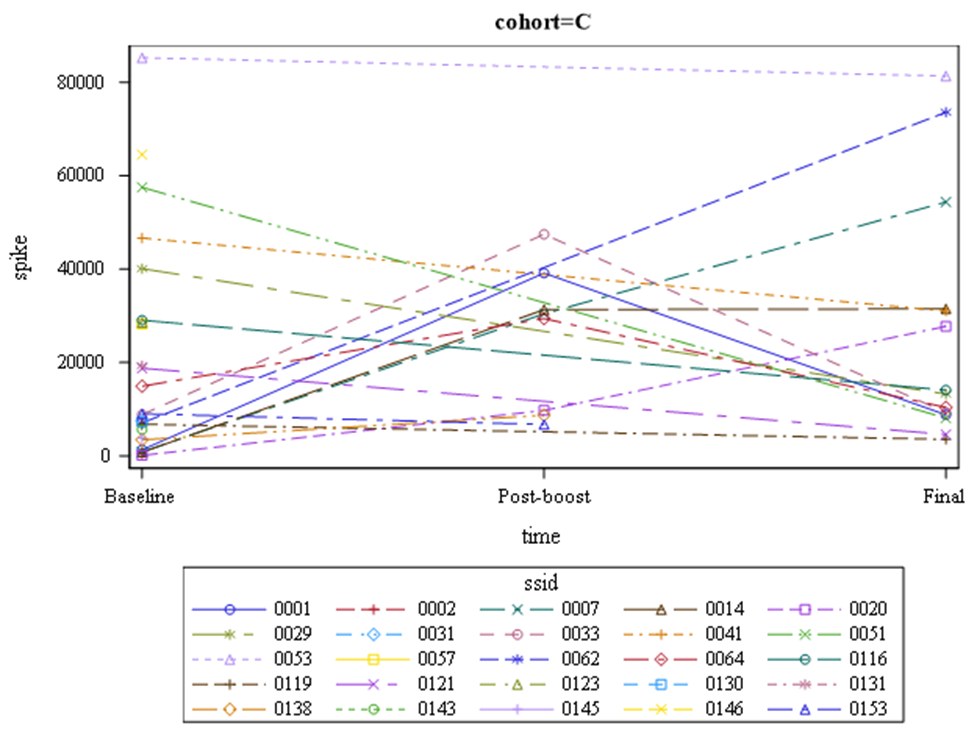


Supp. Fig. S 6. Longitudinal Anti-Spike Titers in Cohort C.

Individual anti-S antibody trajectories in Cohort C from baseline to post-booster and final assessment. Each line represents one patient, showing variation in antibody responses over time.

**Supplementary Table S1.**

Table S 1. Characteristics of patients in cohort B (hematological cancers under active treatment).

| **Patient** | **Cancer diagnosis** | **Treatment at last vaccination before ICF** | **Treatment at booster vaccination** | **Treatment at final assessment** |
| --- | --- | --- | --- | --- |
| 0019 | Sézary Lymphoma | Mogamulizumab | Brentuximab Vedotin | Chemotherapy |
| 0024 | Follicular Lymphoma | Rituximab, cyclophosphamide, doxorubicin, vincristine, prednisone | Booster not done | Rituximab |
| 0025 | Hodgkin Lymphoma | Brentuximab Vedotin | Brentuximab Vedotin | Brentuximab Vedotin |
| 0026 | Chronic Lymphocytic Leukemia | Ibrutinib | Ibrutinib | Sample not collected |
| 0076 | Essential Thrombocythemia | Hydroxycarbamid | Hydroxycarbamid | Sample not collected |
| 0080 | Acute Lymphoblastic Leukemia, in remission | Methotrexate, Vincristine, Mercaptopurine | Methotrexate, Mercaptopurine | Sample not collected |
| 0091 | Acute Myeloid Leukemia, in remission | Venetoclax, Azacitidine | None | Sample not collected |
| 0133 | Multiple Myeloma | Daratumumab, Lenalidomide | Booster not done | Daratumumab, Lenalidomide |
| 0149 | Multiple Myeloma | Daratumumab, Lenalidomide | Daratumumab, Lenalidomide | Sample not collected |

**Supplementary Table S2.**

Table S 2. Absolute titers levels of anti-SARS-CoV-2 spike at pre-booster assessment

|  | | | | | | |
| --- | --- | --- | --- | --- | --- | --- |
|  | cohort | | | |  | |
|  | A.1-2 | A.3 | B | C | Total | P-value |
| **spike_pre_booster** |  |  |  |  |  | 0.5062^1^ |
| N | 2 | 2 | 1 | 4 | 9 |  |
| Mean (SD) | 19880.5 (27087.14) | 26121.0 (7157.33) | 8531.0 (.) | 7076.5 (3245.86) | 14315.6 (13230.17) |  |
| Median (IQR) | 19880.5 (727.0, 39034.0) | 26121.0 (21060.0, 31182.0) | 8531.0 (8531.0, 8531.0) | 7535.0 (4659.5, 9493.5) | 8592.0 (6478.0, 21060.0) |  |
| Range | 727.0, 39034.0 | 21060.0, 31182.0 | 8531.0, 8531.0 | 2841.0, 10395.0 | 727.0, 39034.0 |  |
| Missing | 56 | 20 | 8 | 22 | 106 |  |
|  |  |  |  |  |  |  |
| **nucleo_pre_booster**, n (%) |  |  |  |  |  | 0.8095^2^ |
| NonReac | 1 (50.0%) | 0 (0.0%) | 0 (0.0%) | 2 (50.0%) | 3 (33.3%) |  |
| Reac | 1 (50.0%) | 2 (100.0%) | 1 (100.0%) | 2 (50.0%) | 6 (66.7%) |  |
| Missing | 56 | 20 | 8 | 22 | 106 |  |
|  |  |  |  |  |  |  |
| ^1^Kruskal-Wallis p-value; ^2^Fisher Exact p-value.  IQR, interquartile range; N, number; NonReac, non-reactive; Reac, reactive; SD, standard deviation. | | | | | | |

**Supplementary Table S3.**

Table S 3. Test statistics of paired comparisons of anti-Spike antibody titers between study timepoints (Wilcoxon signed-rank).

| Cohort | Baseline vs post-boost | Post-boost vs final assessment | Baseline vs final assessment |
| --- | --- | --- | --- |
| A.1-2 | P-value <0.001 | P-value 0.006 | P-value 0.31 |
| A.3 | P-value 0.02 | P-value 0.008 | P-value 0.64 |
| B | Not performed due to limited paired samples | | |
| C | P-value 0.004 | P-value 0.25 | P-value 0.72 |
|  | | | |

**Supplementary Table S4.**

Table S 4. Anti-SARS-CoV-2 spike at baseline according to nucleocapsid status in the different cohorts

|  | | nucleo_baseline | |  | |
| --- | --- | --- | --- | --- | --- |
| cohort |  | NonReac | Reac | Total | P-value |
| A.1-2 |  | (N=22) | (N=33) | (N=55) |  |
|  | **spike_baseline** |  |  |  | <.0001^1^ |
|  | N | 22 | 33 | 55 |  |
|  | Mean (SD) | 6583.5 (9264.49) | 29858.7 (26562.07) | 20548.6 (24164.08) |  |
|  | Median (IQR) | 3182.0 (961.0, 8869.0) | 19193.0 (11205.0, 42893.0) | 11205.0 (3310.0, 30506.0) |  |
|  | Range | 583.0, 38010.0 | 105.0, 95904.0 | 105.0, 95904.0 |  |
|  | Missing | 0 | 0 | 0 |  |
| A.3 |  | (N=9) | (N=8) | (N=17) |  |
|  | **spike_baseline** |  |  |  | 0.1489^1^ |
|  | N | 9 | 8 | 17 |  |
|  | Mean (SD) | 2740.3 (2640.04) | 25297.9 (32999.04) | 13355.6 (24790.91) |  |
|  | Median (IQR) | 3197.0 (202.0, 4766.0) | 20367.0 (340.5, 30523.5) | 3875.0 (202.0, 13863.0) |  |
|  | Range | 5.5, 7115.0 | 121.0, 99800.0 | 5.5, 99800.0 |  |
|  | Missing | 0 | 0 | 0 |  |
| B |  | (N=7) | (N=2) | (N=9) |  |
|  | **spike_baseline** |  |  |  | 0.0404^1^ |
|  | N | 7 | 2 | 9 |  |
|  | Mean (SD) | 762.5 (1106.12) | 21127.0 (14992.08) | 5288.0 (10471.46) |  |
|  | Median (IQR) | 246.0 (52.0, 1699.0) | 21127.0 (10526.0, 31728.0) | 330.0 (107.0, 2888.0) |  |
|  | Range | 15.6, 2888.0 | 10526.0, 31728.0 | 15.6, 31728.0 |  |
|  | Missing | 0 | 0 | 0 |  |
| C |  | (N=12) | (N=13) | (N=25) |  |
|  | **spike_baseline** |  |  |  | 0.0002^1^ |
|  | N | 12 | 13 | 25 |  |
|  | Mean (SD) | 4312.5 (3498.44) | 34141.7 (23859.46) | 19823.7 (22838.34) |  |
|  | Median (IQR) | 4533.5 (816.5, 7277.0) | 28582.0 (18766.0, 46633.0) | 8828.0 (3785.0, 28582.0) |  |
|  | Range | 90.9, 9017.0 | 3785.0, 85242.0 | 90.9, 85242.0 |  |
|  | Missing | 0 | 0 | 0 |  |
| ^1^Kruskal-Wallis p-value.  IQR, interquartile range; N, number; NonReac, non-reactive; Reac, reactive; SD, standard deviation. | | | | | |

**Supplementary Table S5.**

Table S 5. Anti-SARS-CoV-2 spike at post-booster according to nucleocapsid status in the different cohorts

|  | | nucleo_post_booster | |  | |
| --- | --- | --- | --- | --- | --- |
| Cohort_booster |  | NonReac | Reac | Total | P-value |
| A.1-2 |  | (N=14) | (N=10) | (N=24) |  |
|  | **spike_post_booster** |  |  |  | 0.0695^1^ |
|  | N | 14 | 10 | 24 |  |
|  | Mean (SD) | 34734.6 (18079.41) | 58364.0 (40922.48) | 44580.2 (31331.41) |  |
|  | Median (IQR) | 29992.0 (19394.0, 41522.0) | 49899.5 (35162.0, 76371.0) | 35200.5 (21847.5, 62998.5) |  |
|  | Range | 17515.0, 69259.0 | 9822.0, 155332.0 | 9822.0, 155332.0 |  |
|  | Missing | 0 | 0 | 0 |  |
| A.3 |  | (N=6) | (N=6) | (N=12) |  |
|  | **spike_post_booster** |  |  |  | 0.0104^1^ |
|  | N | 6 | 6 | 12 |  |
|  | Mean (SD) | 9689.5 (12384.94) | 58941.3 (34546.94) | 34315.4 (35690.11) |  |
|  | Median (IQR) | 3665.5 (380.0, 20444.0) | 62818.5 (34317.0, 91697.0) | 25094.0 (3665.5, 62818.5) |  |
|  | Range | 238.0, 29744.0 | 5572.0, 96425.0 | 238.0, 96425.0 |  |
|  | Missing | 0 | 0 | 0 |  |
| B |  | (N=4) | (N=2) | (N=6) |  |
|  | **spike_post_booster** |  |  |  | 1.0000^1^ |
|  | N | 4 | 2 | 6 |  |
|  | Mean (SD) | 6119.0 (10096.91) | 14185.0 (19790.50) | 8807.7 (12524.01) |  |
|  | Median (IQR) | 1503.5 (355.5, 11882.5) | 14185.0 (191.0, 28179.0) | 1503.5 (287.0, 21182.0) |  |
|  | Range | 287.0, 21182.0 | 191.0, 28179.0 | 191.0, 28179.0 |  |
|  | Missing | 0 | 0 | 0 |  |
| C |  | (N=7) | (N=1) | (N=8) |  |
|  | **spike_post_booster** |  |  |  | 0.8273^1^ |
|  | N | 7 | 1 | 8 |  |
|  | Mean (SD) | 24775.7 (16360.67) | 29401.0 (.) | 25353.9 (15235.06) |  |
|  | Median (IQR) | 30414.0 (8699.0, 39142.0) | 29401.0 (29401.0, 29401.0) | 29907.5 (9210.5, 35220.5) |  |
|  | Range | 6710.0, 47444.0 | 29401.0, 29401.0 | 6710.0, 47444.0 |  |
|  | Missing | 0 | 0 | 0 |  |
| ^1^Kruskal-Wallis p-value.  IQR, interquartile range; N, number; NonReac, non-reactive; Reac, reactive; SD, standard deviation. | | | | | |

**Supplementary Table S6.**

Table S 6. Anti-SARS-CoV-2 spike at final assessment according to nucleocapsid status in the different cohorts.

|  | | nucleo_final | |  | |
| --- | --- | --- | --- | --- | --- |
| Cohort_booster |  | NonReac | Reac | Total | P-value |
| A.1-2 |  | (N=15) | (N=15) | (N=30) |  |
|  | **spike_final** |  |  |  | 0.0020^1^ |
|  | N | 15 | 15 | 30 |  |
|  | Mean (SD) | 7856.8 (6534.53) | 23391.0 (14740.26) | 15623.9 (13708.15) |  |
|  | Median (IQR) | 5368.0 (2016.0, 15297.0) | 24628.0 (8967.0, 33961.0) | 10105.5 (5302.0, 24628.0) |  |
|  | Range | 1389.0, 19264.0 | 1296.0, 54691.0 | 1296.0, 54691.0 |  |
|  | Missing | 0 | 0 | 0 |  |
| A.3 |  | (N=2) | (N=6) | (N=8) |  |
|  | **spike_final** |  |  |  | 0.0956^1^ |
|  | N | 2 | 6 | 8 |  |
|  | Mean (SD) | 2053.0 (2080.31) | 14048.8 (13730.28) | 11049.9 (12888.42) |  |
|  | Median (IQR) | 2053.0 (582.0, 3524.0) | 10205.0 (5244.0, 17866.0) | 7719.5 (2333.5, 14040.5) |  |
|  | Range | 582.0, 3524.0 | 1143.0, 39630.0 | 582.0, 39630.0 |  |
|  | Missing | 0 | 0 | 0 |  |
| B |  | (N=2) | (N=2) | (N=4) |  |
|  | **spike_final** |  |  |  | 1.0000^1^ |
|  | N | 2 | 2 | 4 |  |
|  | Mean (SD) | 3941.0 (2364.57) | 3685.0 (5045.91) | 3813.0 (3220.66) |  |
|  | Median (IQR) | 3941.0 (2269.0, 5613.0) | 3685.0 (117.0, 7253.0) | 3941.0 (1193.0, 6433.0) |  |
|  | Range | 2269.0, 5613.0 | 117.0, 7253.0 | 117.0, 7253.0 |  |
|  | Missing | 0 | 0 | 0 |  |
| C |  | (N=4) | (N=10) | (N=14) |  |
|  | **spike_final** |  |  |  | 0.0109^1^ |
|  | N | 4 | 10 | 14 |  |
|  | Mean (SD) | 6549.0 (2913.67) | 34528.8 (26519.87) | 26534.6 (25708.38) |  |
|  | Median (IQR) | 6684.0 (4058.5, 9039.5) | 29374.5 (13238.0, 54363.0) | 13659.0 (8792.0, 31475.0) |  |
|  | Range | 3541.0, 9287.0 | 8107.0, 81334.0 | 3541.0, 81334.0 |  |
|  | Missing | 0 | 0 | 0 |  |
| ^1^Kruskal-Wallis p-value.  IQR, interquartile range; N, number; NonReac, non-reactive; Reac, reactive; SD, standard deviation. | | | | | |

**Supplementary Table S7-14. Subgroup analysis differences according to nucleocapsid status**

Table S 7. Subgroup analyses by age, anti-SARS-CoV-2 spike at baseline.

|  | | ageclass | | |  | |
| --- | --- | --- | --- | --- | --- | --- |
| nucleo_baseline | | 18-55 | 56-75 | >75 | Total | p-value |
| NonReac | **spike_baseline** |  |  |  |  | 0.6038^1^ |
|  | N | 19 | 28 | 3 | 50 |  |
|  | Mean (SD) | 4004.0 (5521.98) | 3805.5 (4359.54) | 14652.7 (20400.11) | 4531.7 (6733.56) |  |
|  | Median (IQR) | 1297.0  (383.0, 7074.0) | 2971.0  (889.0, 4860.5) | 5618.0  (330.0, 38010.0) | 2864.5  (700.0, 5618.0) |  |
|  | Range | 5.5, 22072.0 | 90.9, 21402.0 | 330.0, 38010.0 | 5.5, 38010.0 |  |
| Reac | **spike_baseline** |  |  |  |  | 0.4259^1^ |
|  | N | 14 | 37 | 5 | 56 |  |
|  | Mean (SD) | 24193.9 (24219.07) | 32854.5 (27315.58) | 23897.0 (24330.61) | 29889.6 (26220.50) |  |
|  | Median (IQR) | 17336.0  (7961.0, 30506.0) | 22468.0  (12322.0, 46633.0) | 13863.0  (11380.0, 31728.0) | 20800.0  (10865.5, 41482.5) |  |
|  | Range | 121.0, 95904.0 | 125.0, 99800.0 | 105.0, 62409.0 | 105.0, 99800.0 |  |
| ^1^Kruskal-Wallis p-value; IQR, interquartile range; N, number; NonReac, non-reactive; Reac, reactive; SD, standard deviation. | | | | | | |

Table S 8. Subgroup analyses by age, anti-SARS-CoV-2 spike at post-booster.

|  | | ageclass | | |  |  |
| --- | --- | --- | --- | --- | --- | --- |
| nucleo_post_booster | | 18-55 | 56-75 | >75 | Total | p-value |
| NonReac | **spike_post_booster** |  |  |  |  | 0.3205^1^ |
|  | N | 11 | 19 | 1 | 31 |  |
|  | Mean (SD) | 29337.9 (23014.19) | 21948.8 (16506.91) | 2583.0 (.) | 23946.1 (19194.70) |  |
|  | Median (IQR) | 29744.0  (6710.0, 41522.0) | 20444.0  (8699.0, 30473.0) | 2583.0  (2583.0, 2583.0) | 20503.0  (6710.0, 33563.0) |  |
|  | Range | 380.0, 69259.0 | 238.0, 61899.0 | 2583.0, 2583.0 | 238.0, 69259.0 |  |
| Reac | **spike_post_booster** |  |  |  |  | 0.9326^1^ |
|  | N | 6 | 12 | 1 | 19 |  |
|  | Mean (SD) | 43601.2 (21868.23) | 56837.4 (45126.31) | 51403.0 (.) | 52371.5 (37633.54) |  |
|  | Median (IQR) | 42034.0  (34317.0, 64366.0) | 48216.5  (25685.5, 85476.5) | 51403.0  (51403.0, 51403.0) | 48396.0  (28179.0, 76371.0) |  |
|  | Range | 9822.0, 69034.0 | 191.0, 155332.0 | 51403.0, 51403.0 | 191.0, 155332.0 |  |
| ^1^Kruskal-Wallis p-value; IQR, interquartile range; N, number; NonReac, non-reactive; Reac, reactive; SD, standard deviation. | | | | | | |

Table S 9. Subgroup analyses by age, anti-SARS-CoV-2 spike at final assessment.

|  |  | ageclass | | |  |  |
| --- | --- | --- | --- | --- | --- | --- |
| nucleo_final |  | 18-55 | 56-75 | >75 | Total | p-value |
| NonReac | **spike_final** |  |  |  |  | 0.9509^1^ |
|  | N | 11 | 12 | 0 | 23 |  |
|  | Mean (SD) | 7213.9 (6598.21) | 6390.3 (4975.82) | - | 6784.2 (5687.32) |  |
|  | Median (IQR) | 4836.0  (2269.0, 15297.0) | 5335.0  (2778.5, 9039.5) | - | 5302.0  (2269.0, 9287.0) |  |
|  | Range | 582.0, 18292.0 | 1389.0, 19264.0 | - | 582.0, 19264.0 |  |
| Reac | **spike_final** |  |  |  |  | 0.2501^1^ |
|  | N | 6 | 25 | 2 | 33 |  |
|  | Mean (SD) | 28027.7 (25025.84) | 24139.2 (19510.77) | 8085.0 (1176.63) | 23873.2 (20056.79) |  |
|  | Median (IQR) | 25813.5  (10195.0, 31475.0) | 17866.0  (10215.0, 33961.0) | 8085.0  (7253.0, 8917.0) | 17866.0  (8967.0, 31475.0) |  |
|  | Range | 1296.0, 73573.0 | 117.0, 81334.0 | 7253.0, 8917.0 | 117.0, 81334.0 |  |
| ^1^Kruskal-Wallis p-value; IQR, interquartile range; N, number; NonReac, non-reactive; Reac, reactive; SD, standard deviation. | | | | | | |

Table S 10. Subgroup analyses by sex, anti-SARS-CoV-2 spike at baseline.

|  | | Sex | |  | |
| --- | --- | --- | --- | --- | --- |
| nucleo_baseline | | F | M | Total | p-value |
| NonReac | **spike_baseline** |  |  |  | 0.0035^1^ |
|  | N | 39 | 11 | 50 |  |
|  | Mean (SD) | 5434.0 (7328.77) | 1332.8 (1907.10) | 4531.7 (6733.56) |  |
|  | Median (IQR) | 3310.0  (933.0, 7115.0) | 330.0  (90.9, 2888.0) | 2864.5  (700.0, 5618.0) |  |
|  | Range | 5.5, 38010.0 | 15.6, 5618.0 | 5.5, 38010.0 |  |
| Reac | **spike_baseline** |  |  |  | 0.5427^1^ |
|  | N | 48 | 8 | 56 |  |
|  | Mean (SD) | 29856.8 (27323.53) | 30086.4 (19726.37) | 29889.6 (26220.50) |  |
|  | Median (IQR) | 19223.0  (10332.5, 41482.5) | 25340.0  (20519.5, 44613.0) | 20800.0  (10865.5, 41482.5) |  |
|  | Range | 105.0, 99800.0 | 556.0, 59190.0 | 105.0, 99800.0 |  |
| ^1^Kruskal-Wallis p-value; IQR, interquartile range; N, number; NonReac, non-reactive; Reac, reactive; SD, standard deviation. | | | | | |

Table S 11. Subgroup analyses by sex, anti-SARS-CoV-2 spike at post-booster.

|  | | sex | |  | |
| --- | --- | --- | --- | --- | --- |
| nucleo_post_booster | | F | M | Total | P-value |
| NonReac | **spike_post_booster** |  |  |  | 0.0532^1^ |
|  | N | 26 | 5 | 31 |  |
|  | Mean (SD) | 26846.7 (18982.66) | 8863.0 (13117.88) | 23946.1 (19194.70) |  |
|  | Median (IQR) | 24611.5  (17515.0, 35239.0) | 2583.0 (424.0, 9722.0) | 20503.0 (6710.0, 33563.0) |  |
|  | Range | 238.0, 69259.0 | 287.0, 31299.0 | 238.0, 69259.0 |  |
| Reac | **spike_post_booster** |  |  |  | 0.6892^1^ |
|  | N | 15 | 4 | 19 |  |
|  | Mean (SD) | 51520.4 (38355.15) | 55563.3 (40190.53) | 52371.5 (37633.54) |  |
|  | Median (IQR) | 35672.0  (28179.0, 76371.0) | 62818.5 (30731.0, 80395.5) | 48396.0 (28179.0, 76371.0) |  |
|  | Range | 5572.0, 155332.0 | 191.0, 96425.0 | 191.0, 155332.0 |  |
| ^1^Kruskal-Wallis p-value; IQR, interquartile range; N, number; NonReac, non-reactive; Reac, reactive; SD, standard deviation. | | | | | |

Table S 12. Subgroup analyses by sex, anti-SARS-CoV-2 spike at final assessment.

|  | | sex | |  | |
| --- | --- | --- | --- | --- | --- |
| nucleo_final | | F | M | Total | p-value |
| NonReac | **spike_final** |  |  |  | 0.8711^1^ |
|  | N | 19 | 4 | 23 |  |
|  | Mean (SD) | 7038.2 (6120.69) | 5577.5 (3173.95) | 6784.2 (5687.32) |  |
|  | Median (IQR) | 5302.0 (2016.0, 9287.0) | 5094.5 (3422.5, 7732.5) | 5302.0 (2269.0, 9287.0) |  |
|  | Range | 582.0, 19264.0 | 2269.0, 9852.0 | 582.0, 19264.0 |  |
| Reac | **spike_final** |  |  |  | 0.4552^1^ |
|  | N | 27 | 6 | 33 |  |
|  | Mean (SD) | 24944.8 (20968.59) | 19051.2 (15940.83) | 23873.2 (20056.79) |  |
|  | Median (IQR) | 17866.0 (10195.0, 33961.0) | 17916.0 (7253.0, 31475.0) | 17866.0 (8967.0, 31475.0) |  |
|  | Range | 1143.0, 81334.0 | 117.0, 39630.0 | 117.0, 81334.0 |  |
| ^1^Kruskal-Wallis p-value; IQR, interquartile range; N, number; NonReac, non-reactive; Reac, reactive; SD, standard deviation. | | | | | |

Table S 13. Subgroup analyses by BMI classification, anti-SARS-CoV-2 spike at baseline.

|  | | overweight_obese | | |  | |
| --- | --- | --- | --- | --- | --- | --- |
| nucleo_baseline | | Missing | no | yes | Total | P-value |
| NonReac | **spike_baseline** |  |  |  |  | 0.4641^1^ |
|  | N | 9 | 19 | 22 | 41 |  |
|  | Mean (SD) | 6718.6 (12141.15) | 4597.1 (5409.30) | 3580.7 (4643.11) | 4051.7 (4974.81) |  |
|  | Median (IQR) | 1699.0 (756.0, 7074.0) | 3197.0 (700.0, 8869.0) | 2706.5 (583.0, 4774.0) | 3054.0 (700.0, 4955.0) |  |
|  | Range | 5.5, 38010.0 | 52.0, 22072.0 | 15.6, 21402.0 | 15.6, 22072.0 |  |
| Reac | **spike_baseline** |  |  |  |  | 0.0046^1^ |
|  | N | 7 | 22 | 27 | 49 |  |
|  | Mean (SD) | 41797.9 (35044.08) | 16911.6 (12992.09) | 37376.9 (28208.29) | 28188.4 (24710.97) |  |
|  | Median (IQR) | 57498.0 (9520.0, 62409.0) | 14829.5 (9098.0, 19327.0) | 30506.0 (15479.0, 54202.0) | 19327.0 (11380.0, 33733.0) |  |
|  | Range | 556.0, 93273.0 | 105.0, 46633.0 | 125.0, 99800.0 | 105.0, 99800.0 |  |
| ^1^Kruskal-Wallis p-value; IQR, interquartile range; N, number; NonReac, non-reactive; Reac, reactive; SD, standard deviation. | | | | | | |

Table S 14. Subgroup analyses by BMI classification, anti-SARS-CoV-2 spike at post-booster.

|  | | overweight_obese | | |  | |
| --- | --- | --- | --- | --- | --- | --- |
| nucleo_post_booster | | Missing | no | yes | Total | P-value |
| NonReac |  | (N=7) | (N=10) | (N=14) | (N=24) |  |
|  | **spike_post_booster** |  |  |  |  | 0.4124^1^ |
|  | N | 7 | 10 | 14 | 24 |  |
|  | Mean (SD) | 28529.1 (20734.91) | 17778.1 (14386.53) | 26060.2 (21520.44) | 22609.3 (18977.74) |  |
|  | Median (IQR) | 21182.0 (17515.0, 47444.0) | 18492.5 (4174.0, 29744.0) | 28776.0 (8699.0, 31299.0) | 20473.5 (5442.0, 30886.0) |  |
|  | Range | 424.0, 61899.0 | 380.0, 41522.0 | 238.0, 69259.0 | 238.0, 69259.0 |  |
| Reac |  | (N=4) | (N=5) | (N=10) | (N=15) |  |
|  | **spike_post_booster** |  |  |  |  | 0.0864^1^ |
|  | N | 4 | 5 | 10 | 15 |  |
|  | Mean (SD) | 37006.8 (26800.55) | 31700.2 (29372.89) | 68853.1 (39567.07) | 56468.8 (39769.13) |  |
|  | Median (IQR) | 43282.5 (17676.5, 56337.0) | 28179.0 (9822.0, 35672.0) | 66700.0 (34317.0, 91697.0) | 48396.0 (28179.0, 79256.0) |  |
|  | Range | 191.0, 61271.0 | 5572.0, 79256.0 | 23192.0, 155332.0 | 5572.0, 155332.0 |  |
|  | Missing | 0 | 0 | 0 | 0 |  |
| ^1^Kruskal-Wallis p-value; IQR, interquartile range; N, number; NonReac, non-reactive; Reac, reactive; SD, standard deviation. | | | | | | |

**Supplementary Table S15-19. Subgroup analysis for pre-booster assessment.**

Table S 15. Subgroup analysis by age classification, anti-SARS-CoV-2 spike at pre-booster.

|  | ageclass | | |  | |
| --- | --- | --- | --- | --- | --- |
|  | 18-55 | 56-75 | >75 | Total | P-value |
| **spike_pre_booster** |  |  |  |  | 0.1635^1^ |
| N | 2 | 6 | 1 | 9 |  |
| Mean (SD) | 30047.0 (12709.54) | 9725.2 (10972.77) | 10395.0 (.) | 14315.6 (13230.17) |  |
| Median (IQR) | 30047.0  (21060.0, 39034.0) | 7504.5  (2841.0, 8592.0) | 10395.0  (10395.0, 10395.0) | 8592.0  (6478.0, 21060.0) |  |
| Range | 21060.0, 39034.0 | 727.0, 31182.0 | 10395.0, 10395.0 | 727.0, 39034.0 |  |
| Missing | 37 | 62 | 7 | 106 |  |
| **nucleo_pre_booster**, n (%) |  |  |  |  | 0.6429^2^ |
| NonReac | 0 (0.0%) | 3 (50.0%) | 0 (0.0%) | 3 (33.3%) |  |
| Reac | 2 (100.0%) | 3 (50.0%) | 1 (100.0%) | 6 (66.7%) |  |
| Missing | 37 | 62 | 7 | 106 |  |
| ^1^Kruskal-Wallis p-value; ^2^Fisher Exact p-value.  IQR, interquartile range; N, number; NonReac, non-reactive; Reac, reactive; SD, standard deviation. | | | | | |

Table S 16. Subgroup analysis by sex classification, anti-SARS-CoV-2 spike at pre-booster.

|  | sex | |  | |
| --- | --- | --- | --- | --- |
|  | F | M | Total | P-value |
| **spike_pre_booster** |  |  |  | 0.3798^1^ |
| N | 7 | 2 | 9 |  |
| Mean (SD) | 13912.1 (14934.86) | 15727.5 (7541.29) | 14315.6 (13230.17) |  |
| Median (IQR) | 8531.0  (2841.0, 31182.0) | 15727.5  (10395.0, 21060.0) | 8592.0  (6478.0, 21060.0) |  |
| Range | 727.0, 39034.0 | 10395.0, 21060.0 | 727.0, 39034.0 |  |
| Missing | 88 | 18 | 106 |  |
| ^1^Kruskal-Wallis p-value; ^2^Fisher Exact p-value;  IQR, interquartile range; N, number; SD, standard deviation. | | | | |

Table S 17. Subgroup analysis by overweight/obesity, anti-SARS-CoV-2 spike at pre-booster.

|  | overweight_obese | | |  | |
| --- | --- | --- | --- | --- | --- |
|  | Missing | no | yes | Total | P-value |
| **spike_pre_booster** |  |  |  |  | 0.4561^1^ |
| N | 1 | 3 | 5 | 8 |  |
| Mean (SD) | 6478.0 (.) | 13480.0 (15819.17) | 16384.4 (14273.08) | 15295.3 (13790.23) |  |
| Median (IQR) | 6478.0  (6478.0, 6478.0) | 8531.0  (727.0, 31182.0) | 10395.0  (8592.0, 21060.0) | 9493.5  (5686.0, 26121.0) |  |
| Range | 6478.0, 6478.0 | 727.0, 31182.0 | 2841.0, 39034.0 | 727.0, 39034.0 |  |
| Missing | 15 | 43 | 48 | 91 |  |
| **nucleo_pre_booster**, n (%) |  |  |  |  | 1.0000^2^ |
| NonReac | 1 | 1 (33.3%) | 1 (20.0%) | 2 (25.0%) |  |
| Reac | 0 | 2 (66.7%) | 4 (80.0%) | 6 (75.0%) |  |
| Missing | 15 | 43 | 48 | 91 |  |
| ^1^Kruskal-Wallis p-value; ^2^Fisher Exact p-value.  IQR, interquartile range; N, number; NonReac, non-reactive; Reac, reactive; SD, standard deviation. | | | | | |

Table S 18. Subgroup analysis by smoker status, anti-SARS-CoV-2 spike at pre-booster.

|  | smoking | | | |  | |
| --- | --- | --- | --- | --- | --- | --- |
|  | Missing | CURRENT | FORMER | NEVER | Total | P-value |
| **spike_pre_booster** |  |  |  |  |  | 0.1512^1^ |
| N | 1 | 3 | 2 | 3 | 8 |  |
| Mean (SD) | 21060.0 (.) | 5245.3 (4045.39) | 24714.5 (20250.83) | 14205.0 (14981.07) | 13472.5 (13882.80) |  |
| Median (IQR) | 21060.0  (21060.0, 21060.0) | 6478.0  (727.0, 8531.0) | 24714.5  (10395.0, 39034.0) | 8592.0  (2841.0, 31182.0) | 8561.5  (4659.5, 20788.5) |  |
| Range | 21060.0, 21060.0 | 727.0, 8531.0 | 10395.0, 39034.0 | 2841.0, 31182.0 | 727.0, 39034.0 |  |
| Missing | 16 | 14 | 17 | 59 | 90 |  |
| **nucleo_pre_booster**, n (%) |  |  |  |  |  | 0.6786^2^ |
| NonReac | 0 | 2 (66.7%) | 0 (0.0%) | 1 (33.3%) | 3 (37.5%) |  |
| Reac | 1 | 1 (33.3%) | 2 (100.0%) | 2 (66.7%) | 5 (62.5%) |  |
| Missing | 16 | 14 | 17 | 59 | 90 |  |
| ^1^Kruskal-Wallis p-value; ^2^Fisher Exact p-value.  IQR, interquartile range; N, number; NonReac, non-reactive; Reac, reactive; SD, standard deviation. | | | | | | |

Table S 19. Subgroup analysis by absolute lymphocyte count at baseline, anti-SARS-CoV-2 spike at pre-booster.

|  | ALCclass | | |  | |
| --- | --- | --- | --- | --- | --- |
|  | Missing | a. <1000 | b. >=1000 | Total | P-value |
| **spike_pre_booster** |  |  |  |  | 0.5050^1^ |
| N | 1 | 2 | 6 | 8 |  |
| Mean (SD) | 10395.0 (.) | 19856.5 (16016.68) | 13122.0 (14549.52) | 14805.6 (14056.04) |  |
| Median (IQR) | 10395.0  (10395.0, 10395.0) | 19856.5  (8531.0, 31182.0) | 7535.0  (2841.0, 21060.0) | 8561.5  (4659.5, 26121.0) |  |
| Range | 10395.0, 10395.0 | 8531.0, 31182.0 | 727.0, 39034.0 | 727.0, 39034.0 |  |
| Missing | 15 | 21 | 70 | 91 |  |
| **nucleo_pre_booster**, n (%) |  |  |  |  | 0.4643^2^ |
| NonReac | 0 | 0 (0.0%) | 3 (50.0%) | 3 (37.5%) |  |
| Reac | 1 | 2 (100.0%) | 3 (50.0%) | 5 (62.5%) |  |
| Missing | 15 | 21 | 70 | 91 |  |
| ^1^Kruskal-Wallis p-value; ^2^Fisher Exact p-value.  IQR, interquartile range; N, number; NonReac, non-reactive; Reac, reactive; SD, standard deviation. | | | | | |

**Supplementary Table S20.**

Table S 20. Multivariable robust regression model.

| **Multivariable robust regression model** | | | | | |
| --- | --- | --- | --- | --- | --- |
| **Parameter** | **Estimate** | **95% CI** | | **χ2** | **Pr > ChiSq** |
| **Intercept** | 11,389 | 1,322 | 21,456 | 4.92 | 0.027 |
| **Cohorts** |  |  |  |  |  |
| Cohort A.1–2 | Ref | Ref | Ref | Ref | Ref |
| Cohort A.3 | -2,012 | -7,911 | 3,887 | 0.45 | 0.504 |
| Cohort B | -2,716 | -11,346 | 5,914 | 0.38 | 0.537 |
| Cohort C | 2,086 | -3,438 | 7,610 | 0.55 | 0.459 |
| **Anti-Nucleocapsid status at baseline** | | | | | |
| Reactive | Ref | Ref | Ref | Ref | Ref |
| Non-reactive | -13,619 | -18,187 | -9,051 | 34.15 | <0.0001 |
| **Sex** | | | | | |
| Female | Ref | Ref | Ref | Ref | Ref |
| Male | 329 | -5,901 | 6,558 | 0.01 | 0.918 |
| **Cancer status at enrolment ^a^** | | | | | |
| Non-metastatic | Ref | Ref | Ref | Ref | Ref |
| Metastatic | -1,127 | -5,989 | 3,735 | 0.21 | 0.650 |
| **Timing of lase dose** |  |  |  |  |  |
| <6 months | Ref | Ref | Ref | Ref | Ref |
| 6-9 months | -1,879 | -6,994 | 3,237 | 0.52 | 0.472 |
| > 9 months | -26 | -5,462 | 5,410 | 0.00 | 0.993 |
| **No of vaccine prior doses** | | | | | |
| Continuous | 2,420 | -141 | 4,981 | 3.43 | 0.064 |
| **Multivariable regression model with backward variable selection** | | | | | |
| **Parameter** | **Estimate** | **95% CI** | | **χ2** | **Pr > ChiSq** |
| **Intercept** | 8,984 | 993 | 16,974 | 4.86 | 0.028 |
| **Anti-Nucleocapsid status at baseline** | | | | | |
| Reactive | Ref | Ref | Ref | Ref | Ref |
| Non-reactive | -12,197 | -15,974 | -8,420 | 40.07 | <0.0001 |
| **No of vaccine prior doses** | | | | | |
| Continuous | 2,397 | 294 | 4,499 | 4.99 | 0.026 |

Full multivariable robust regression model evaluating associations with baseline anti-SARS-CoV-2 spike antibody titers. All clinical and demographic covariates were included prior to backward selection. Subsequent multivariable robust regression model with backward variable selection.

**Supplementary Table S21.**

Table S 21. Characteristics of patients included in the flow cytometry analysis.

| Patient | Cohort | Sex | Age | Cancer Diagnosis | Treatment at last vaccination before ICF | Treatment at booster vaccination | Baseline | Post-Booster | Final Assessment |
| --- | --- | --- | --- | --- | --- | --- | --- | --- | --- |
| 0002 | C | F | 53 | Breast | 0 | Targeted therapy | Yes | Yes | Yes |
| 0003 | A.3 | F | 50 | Breast | Chemot. ± other agent | Endocrine therapy | Yes | Yes | No |
| 0004 | A.3 | F | 57 | Breast | Chemot. ± other agent | Antibody-drug conjugate | Yes | Yes | Yes |
| 0005 | A.2 | F | 58 | Breast | Endocrine therapy | Endocrine therapy | Yes | Yes | Yes |
| 0006 | A.3 | F | 74 | Lung | 0 | Chemot. ± other agent | Yes | Yes | No |
| 0007 | C | F | 59 | Breast | 0 | 0 | Yes | No | Yes |
| 0008 | A.2 | F | 66 | Breast | Endocrine therapy | Endocrine therapy | Yes | Yes | Yes |
| 0009 | A.3 | F | 48 | Breast | Chemot. ± other agent | Targeted therapy | Yes | Yes | Yes |
| 0010 | A.2 | F | 38 | Breast | Targeted therapy | Targeted therapy | Yes | Yes | Yes |
| 0011 | A.3 | F | 55 | Lung | Chemot. ± other agent | Immunotherapy | Yes | Yes | Yes |
| 0012 | A.3 | F | 32 | Colon | 0 | Chemot. ± other agent | Yes | Yes | Yes |
| 0013 | A.3 | M | 56 | Bladder | Chemot. ± other | Chemot. ± other agent | Yes | Yes | Yes |
| 0014 | C | M | 53 | Lung | 0 | 0 | Yes | Yes | Yes |
| 0015 | A.2 | F | 54 | Breast | Endocrine therapy | Endocrine therapy | Yes | Yes | Yes |
| 0017 | A.2 | M | 51 | Lung | Targeted therapy | Deceased Patient | Yes | Yes | Yes |
| 0018 | A.3 | F | 42 | Breast | 0 | Chemot. ± other agent | Yes | Yes | Yes |
| 0020 | C | M | 70 | Head and Neck | 0 | 0 | Yes | Yes | Yes |
| 0027 | A.2 | F | 72 | Breast | Endocrine therapy | Endocrine therapy | Yes | Yes | Yes |
| 0029 | C | F | 61 | Breast | 0 | 0 | Yes | No | Yes |
| Chemot, chemotherapy; ICF, informed consent form; F, female; M, male. | | | | | | | | | |

**Supplementary Table S22.**

Table S 22. Eligibility criteria for the I-SPARC trial, protocol version 6.0.

| **Eligibility criteria for the I-SPARC trial, protocol version 6.0** |
| --- |
| Inclusion criteria  Subjects must meet all of the following criteria in order to be eligible for this study:   1. Age ≥ 18 years old 2. ECOG performance status ≤ 2 3. Subjects with histologically or cytologically confirmed cancer diagnosis (invasive solid tumour or haematological malignancy)  - undergoing active systemic cancer treatment at the time of the last dose (before ICF signature) of the anti-SARS-CoV-2 mRNA vaccine (such as chemotherapy, immunotherapy, targeted agents, endocrine therapy) in   - non-metastatic/curative setting **or**   - metastatic/palliative setting - or undergoing follow-up after confirmed cancer complete remission without active cancer treatment for the last 12 months at the time of the last dose (before ICF signature) of the anti-SARS-CoV-2 mRNA vaccine.  1. Life expectancy > 6 months 2. Subjects who received at least 2 doses of mRNA platform vaccination against SARS-CoV-2 as per local guidelines, with the last dose being given between 3 and 12 months prior to baseline assessment. 3. Urine/serum pregnancy test negative for all female subjects of childbearing potential within 7 days prior to subject enrolment. 4. Signed Informed Consent form (ICF) obtained prior to any study related procedure. 5. Subject is willing and able to comply with the protocol for the duration of the study including treatment and scheduled visits and examinations. |
| Exclusion criteria  Subjects meeting one of the following criteria are not eligible for this study:   1. Known pregnant and/or lactating women. 2. Subject with a known significant medical, neuro-psychiatric, or surgical condition, currently uncontrolled by treatment, which, in the principal investigator’s opinion, may interfere with completion of the study. 3. Subjects with active diagnosis of acute leukaemia. 4. Subjects treated with bone marrow transplant < 90 days before the last dose of vaccination against SARS-CoV-2 received before ICF signature. 5. Subjects with a known history of HIV infection. 6. COVID-19 infection in the last 28 days prior to subject enrolment. 7. Subjects receiving prolonged and/or high doses of systemic immunosuppressive therapies including corticosteroids during the last 28 days before receiving first dose of vaccination against SARS-CoV-2 and up to subject enrolment. 8. Subjects who, for any reason, did not receive the 2^nd^ dose of the anti-SARS-CoV-2 mRNA vaccine. 9. Subjects that received the 3rd dose of anti-SARS-CoV-2 mRNA vaccine prior to study entry. Exclusion criterion number 9 is only applicable for previous versions of the protocol and is not applicable for protocol version 3.0 and subsequent versions. 10. Subject that received any dose of non-mRNA anti-SARS-CoV-2 vaccine platform. 11. Subjects with a known or suspected history of severe adverse reactions associated with a vaccine and/or with severe allergic reaction to vaccine components or anaphylaxis in the past. 12. Subjects who planned to receive any other licensed vaccines for other indications within 28 days prior to the first booster dose after ICF signature, or who are planning to receive any other vaccine up to 14 days after the first booster dose of the mRNA anti-SARS-CoV-2 vaccine after ICF signature (28 days for live attenuated vaccines). For influenza vaccination, a shorter interval or simultaneous administration is acceptable. 13. Subjects who have planned to receive a booster dose after ICF signature but before the baseline assessment. 14. Subjects who received COVID-19 pre-exposure prophylactic monoclonal antibodies or who have been treated with anti-SARS-CoV-2 monoclonal antibodies or COVID-19 convalescent plasma during the last 6 months before ICF signature. |

**Supplementary Table S23.**

Table S 23. Antibodies used for flow cytometry analysis

| **Name** |  | **Clone** | **Conjugation** | **Firme** | **Reference** | **Applications** |
| --- | --- | --- | --- | --- | --- | --- |
| CD138 |  | 44F9 | APC | Miltenyi Biotec | 130-098-746 | Flow cytometry |
| CD183 | CXCR3 | CEW33D | eFluor 660 | eBioscience | 50-1839-42 | Flow cytometry |
| CD185 | CXCR5 | MU5UBEE | PE | eBioscience | 12-9185-42 | Flow cytometry |
| CD19 |  | REA675 | APC-Vio 770 | Miltenyi Biotec | 130-113-643 | Flow cytometry |
| CD196 | CCR6 | R6H1 | Super Bright 436 | eBioscience | 62-1969-42 | Flow cytometry |
| CD197 | CCR7 | G043H7 | APC-Cyanine7 | BioLegend | 353212 | Flow cytometry |
| CD21 |  | REA940 | FITC | Miltenyi Biotec | 130-115-515 | Flow cytometry |
| CD27 |  | LG.7F9 | PE-Cyanine7 | eBioscience | 25-0271-82 | Flow cytometry |
| CD278 | ICOS | ISA-3 | APC | eBioscience | 17-9948-42 | Flow cytometry |
| CD279 | PD-1 | PD1.3.1.3 | Vio Bright FITC | Miltenyi Biotec | 130-117-681 | Flow cytometry |
| CD38 |  | HB7 | PerCP-eFluor 710 | eBioscience | 46-0388-42 | Flow cytometry |
| CD4 |  | RPA-T4 | Alexa Fluor 700 | eBioscience | 56-0049-42 | Flow cytometry |
| CD45 |  | HI30 | Pacific Oranger | Invitrogen | MHCD4530 | Flow cytometry |
| CD45RA |  | REA562 | PE-Vio770 | Miltenyi Biotec | 130-113-367 | Flow cytometry |
| CD8a |  | RPA-T8 | PE-eFluor610 | eBioscience | 61-0088-42 | Flow cytometry |
| IgD |  | REA740 | PE | Miltenyi Biotec | 130-110-643 | Flow cytometry |
| IgG |  | IS11-3B2.2.3 | VioBlue | Miltenyi Biotec | 130-119-881 | Flow cytometry |
| CD20 |  | 2H7 | AF700 | eBioscience | 56-0209-42 | Flow cytometry |

# **Annex I.**

Evaluability of Patients in the I-SPARC trial.

| Participant  ID | Evaluable? | Evaluable for baseline timepoint? | Booster vaccination on study? | Evaluable for pre-booster timepoint? | Evaluable for post-booster timepoint? | Evaluable for final assessment timepoint? | Explanation |
| --- | --- | --- | --- | --- | --- | --- | --- |
| 0001 | Yes | Yes | Yes | NA | Yes | Yes |  |
| 0002 | Yes | Yes | Yes | NA | Yes | Yes |  |
| 0003 | Yes | Yes | Yes | NA | Yes | No |  |
| 0004 | Yes | Yes | Yes | NA | Yes | Yes |  |
| 0005 | Yes | Yes | Yes | NA | Yes | Yes |  |
| 0006 | Yes | No | Yes | NA | Yes | No | Violation of IC3: Active cancer but no treatment at last vaccination (baseline sample not evaluable); at booster, patient under active treatment (post-booster sample evaluable); pre-booster not applicable and final assessment not collected. |
| 0007 | Yes | Yes | Yes | NA | Yes | Yes |  |
| 0008 | Yes | Yes | Yes | NA | Yes | Yes |  |
| 0009 | Yes | Yes | Yes | NA | Yes | Yes |  |
| 0010 | Yes | Yes | Yes | NA | Yes | Yes |  |
| 0011 | Yes | Yes | Yes | NA | Yes | Yes |  |
| 0012 | Yes | No | Yes | NA | Yes | Yes | Violation of IC3: No cancer diagnosis at last vaccination (baseline sample not evaluable); at booster, patient under active treatment (post-booster and final assessment samples evaluable); pre-booster sample not applicable. |
| 0013 | Yes | Yes | Yes | NA | Yes | Yes |  |
| 0014 | Yes | Yes | Yes | NA | Yes | Yes |  |
| 0015 | Yes | Yes | Yes | NA | Yes | Yes |  |
| 0016 | No | No | Yes | No | No | No | Violation of EC3: diagnosis of active acute leukemia. |
| 0017 | Yes | Yes | No | NA | NA | NA |  |
| 0018 | Yes | No | Yes | NA | Yes | Yes | Violation of IC3: No cancer diagnosis at last vaccination (baseline sample not evaluable); at booster, patient under active treatment (post-booster and final assessment samples evaluable); pre-booster sample not applicable. |
| 0019 | Yes | Yes | Yes | NA | Yes | Yes |  |
| 0020 | Yes | Yes | Yes | NA | Yes | Yes |  |
| 0021 | Yes | Yes | Yes | NA | Yes | Yes |  |
| 0023 | Yes | Yes | Yes | NA | Yes | Yes |  |
| 0024 | Yes | Yes | No | NA | NA | Yes |  |
| 0025 | Yes | Yes | Yes | NA | NA | Yes |  |
| 0026 | Yes | Yes | Yes | NA | Yes | No |  |
| 0027 | Yes | Yes | Yes | NA | Yes | Yes |  |
| 0028 | No | No | No | No | No | No | Violation of IC3: Active cancer at last vaccination but no active treatment; No booster vaccination on study. No evaluable samples. |
| 0029 | Yes | Yes | Yes | Yes | No | Yes |  |
| 0030 | Yes | Yes | No | NA | NA | NA |  |
| 0031 | Yes | Yes | No | NA | NA | NA |  |
| 0032 | Yes | Yes | No | NA | NA | Yes |  |
| 0033 | Yes | Yes | Yes | Yes | Yes | Yes |  |
| 0034 | Yes | Yes | Yes | NA | Yes | Yes |  |
| 0035 | Yes | Yes | No | NA | NA | NA |  |
| 0036 | Yes | Yes | No | NA | NA | NA |  |
| 0037 | No | No | No | No | No | No | Violation of IC3: Active cancer at last vaccination but no active treatment; No booster vaccination on study. No evaluable samples. |
| 0038 | Yes | Yes | Yes | NA | Yes | No |  |
| 0039 | Yes | Yes | No | NA | NA | NA |  |
| 0040 | No | No | No | No | No | No | Violation of IC3: No cancer diagnosis at last vaccination (baseline sample not evaluable); No booster vaccination on study (no other evaluable samples). |
| 0041 | Yes | Yes | No | NA | NA | Yes |  |
| 0042 | No | No | No | No | No | No | Violation of IC3: No cancer diagnosis at last vaccination (baseline sample not evaluable); No booster vaccination on study (no other evaluable samples). |
| 0043 | Yes | Yes | No | NA | NA | Yes |  |
| 0044 | Yes | Yes | Yes | NA | Yes | Yes |  |
| 0045 | Yes | Yes | No | NA | NA | NA |  |
| 0046 | Yes | Yes | No | NA | NA | Yes |  |
| 0047 | Yes | Yes | Yes | NA | Yes | Yes |  |
| 0048 | Yes | Yes | Yes | NA | Yes | No |  |
| 0049 | Yes | No | Yes | NA | Yes | Yes | Violation of IC3: No cancer diagnosis at last vaccination (baseline sample not evaluable); at booster, patient under active treatment (post-booster and final assessment samples evaluable); pre-booster sample not applicable. |
| 0050 | No | No | No | No | No | No | Violation of IC3: No cancer diagnosis at last vaccination (baseline sample not evaluable); No booster vaccination on study (no other evaluable samples). |
| 0051 | Yes | Yes | Yes | NA | NA | Yes |  |
| 0052 | No | No | No | No | No | No | Violation of IC3: No cancer diagnosis at last vaccination (baseline sample not evaluable); No booster vaccination on study (no other evaluable samples). |
| 0053 | Yes | Yes | Yes | NA | Yes | Yes |  |
| 0054 | Yes | No | Yes | No | Yes | NA | Violation of IC3: No cancer diagnosis at last vaccination (baseline and pre-booster samples not evaluable);; at booster, patient under active treatment (post-booster sample evaluable); Final assessmement sample not collected. |
| 0055 | Yes | Yes | Yes | NA | Yes | No |  |
| 0056 | Yes | Yes | Yes | NA | Yes | No |  |
| 0057 | Yes | Yes | No | NA | NA | NA |  |
| 0058 | Yes | Yes | No | NA | NA | Yes |  |
| 0059 | Yes | Yes | Yes | NA | Yes | No |  |
| 0060 | Yes | Yes | No | NA | NA | NA |  |
| 0061 | Yes | Yes | Yes | Yes | Yes | NA |  |
| 0062 | Yes | Yes | No | NA | NA | Yes |  |
| 0063 | Yes | Yes | No | NA | NA | NA |  |
| 0064 | Yes | Yes | Yes | NA | Yes | Yes |  |
| 0065 | No | No | No | No | No | No | Violation of IC3: Active cancer at last vaccination but no active treatment; No booster vaccination on study. No evaluable samples. |
| 0066 | No | No | Yes | No | No | No | Violation of IC3: No cancer diagnosis at last vaccination (baseline sample not evaluable); Cancer diagnosis under active treatment at booster vaccination on study, patient refused blood collection (no other evaluable samples). |
| 0067 | No | No | No | No | No | No | Violation of IC3: Diagnosis of active cancer at last vaccination but no active treatment (baseline sample not evaluable); No booster vaccination on study (no other samples evaluable). |
| 0068 | No | No | No | No | No | No |  |
| 0069 | Yes | Yes | No | NA | NA | NA |  |
| 0070 | Yes | Yes | Yes | NA | NA | NA |  |
| 0071 | Yes | Yes | Yes | NA | NA | NA |  |
| 0072 | Yes | Yes | Yes | NA | NA | NA |  |
| 0073 | No | No | Yes | No | No | No | Violation of IC3: No cancer diagnosis at last vaccination (baseline sample not evaluable); Cancer diagnosis under treatment at booster vaccination, but no samples collected (no other evaluable samples). |
| 0074 | No | No | Yes | No | No | No | Violation of EC10: Receipt of any dose of a non-mRNA anti-SARS-CoV-2 vaccine. |
| 0075 | Yes | Yes | Yes | NA | NA | NA |  |
| 0076 | Yes | Yes | Yes | NA | Yes | NA |  |
| 0077 | No | No | No | No | No | No | Violation of EC10: Receipt of any dose of a non-mRNA anti-SARS-CoV-2 vaccine. |
| 0078 | Yes | Yes | Yes | NA | Yes | NA |  |
| 0079 | No | No | No | No | No | No | Violation of EC10: Receipt of any dose of a non-mRNA anti-SARS-CoV-2 vaccine. |
| 0080 | Yes | Yes | Yes | NA | Yes | NA |  |
| 0081 | No | No | Yes | No | No | No | Violation of IC3: Diagnosis of active cancer at last vaccination, but no active treatment (baseline sample not evaluable); Booster vaccination on study but no samples collected (no other evaluable samples). |
| 0082 | No | No | Yes | No | No | No | Violation of EC10: Receipt of any dose of a non-mRNA anti-SARS-CoV-2 vaccine. |
| 0083 | Yes | Yes | Yes | NA | NA | Yes |  |
| 0084 | No | No | Yes | No | No | No | Violation of EC10: Receipt of any dose of a non-mRNA anti-SARS-CoV-2 vaccine. |
| 0085 | No | No | Yes | No | No | No | Violation of EC10: Receipt of any dose of a non-mRNA anti-SARS-CoV-2 vaccine. |
| 0086 | Yes | Yes | Yes | NA | Yes | NA |  |
| 0087 | Yes | Yes | Yes | NA | NA | NA |  |
| 0088 | Yes | No | Yes | NA | Yes | NA | Violation of IC3: No cancer diagnosis at last vaccination (baseline sample not evaluable); at booster, patient under active treatment (post-booster sample evaluable); pre-booster not applicable and final assessment samples not collected. |
| 0089 | Yes | Yes | No | NA | Yes | Yes |  |
| 0090 | Yes | No | Yes | Yes | Yes | NA | Violation of IC3: No cancer diagnosis at last vaccination (baseline sample not evaluable); at booster, patient under active treatment (pre- and post-booster samples evaluable); final assessment sample not collected. |
| 0091 | Yes | Yes | Yes | Yes | Yes | NA |  |
| 0092 | Yes | Yes | Yes | NA | Yes | NA |  |
| 0093 | Yes | No | Yes | NA | Yes | Yes | Violation of IC3: No cancer diagnosis at last vaccination (baseline sample not evaluable); at booster, patient under active treatment (post-booster and final assessment samples evaluable); pre-booster sample not applicable. |
| 0094 | No | No | No | No | No | No | Violation of IC3: No cancer diagnosis at last vaccination (baseline sample not evaluable); No booster vaccination on study (no other evaluable samples). |
| 0095 | Yes | Yes | No | NA | NA | NA |  |
| 0096 | Yes | Yes | No | NA | NA | Yes |  |
| 0097 | No | No | No | No | No | No | No available blood samples. |
| 0098 | No | No | No | No | No | No |  |
| 0099 | No | No | No | No | No | No | Violation of EC10: Receipt of any dose of a non-mRNA anti-SARS-CoV-2 vaccine. |
| 0100 | Yes | Yes | No | NA | NA | NA |  |
| 0101 | Yes | Yes | No | NA | NA | NA |  |
| 0102 | No | No | No | No | No | No | Violation of IC3: Diagnosis of active cancer at last vaccination but no active treatment (baseline sample not evaluable); No booster vaccination on study (no other samples evaluable). |
| 0103 | Yes | Yes | No | NA | NA | Yes |  |
| 0104 | Yes | Yes | No | NA | NA | Yes |  |
| 0105 | Yes | Yes | No | NA | NA | Yes |  |
| 0106 | Yes | Yes | No | NA | NA | Yes |  |
| 0107 | Yes | Yes | No | NA | NA | Yes |  |
| 0108 | Yes | Yes | No | NA | NA | Yes |  |
| 0109 | No | No | No | No | No | No | Violation of EC10: Receipt of any dose of a non-mRNA anti-SARS-CoV-2 vaccine. |
| 0110 | No | No | No | NA | NA | No | Violation of IC3: Cancer diagnosis at last vaccination, but under remission for <12months (baseline sample not evaluable); No booster vaccination on study (no other evaluable samples). |
| 0111 | No | No | No | No | No | No | Violation of EC10: Receipt of any dose of a non-mRNA anti-SARS-CoV-2 vaccine. |
| 0112 | Yes | Yes | No | NA | NA | Yes |  |
| 0113 | Yes | Yes | No | NA | NA | Yes |  |
| 0114 | Yes | Yes | No | NA | NA | Yes |  |
| 0115 | Yes | Yes | No | NA | NA | NA |  |
| 0116 | Yes | Yes | No | NA | NA | Yes |  |
| 0117 | No | No | No | No | No | No | Violation of IC3: Diagnosis of active cancer at last vaccination but no active treatment (baseline sample not evaluable); No booster vaccination on study (no other samples evaluable). |
| 0118 | Yes | Yes | No | NA | NA | NA |  |
| 0119 | Yes | Yes | No | NA | NA | Yes |  |
| 0120 | Yes | Yes | Yes | NA | NA | NA |  |
| 0121 | Yes | Yes | No | NA | NA | Yes |  |
| 0122 | No | No | No | No | No | No | Violation of IC3: No cancer diagnosis at last vaccination (baseline sample not evaluable); No booster vaccination on study (no other evaluable samples). |
| 0123 | Yes | Yes | No | NA | NA | NA |  |
| 0124 | No | No | Yes | No | No | No | Violation of IC3: Diagnosis of active cancer at last vaccination but no active treatment (baseline sample not evaluable); No booster vaccination on study (no other samples evaluable). |
| 0125 | No | No | No | No | No | No | Violation of IC3: No cancer diagnosis at last vaccination (baseline sample not evaluable); No booster vaccination on study (no other evaluable samples). |
| 0126 | No | No | No | No | No | No |  |
| 0127 | Yes | Yes | No | NA | NA | NA |  |
| 0128 | Yes | Yes | No | NA | NA | Yes |  |
| 0129 | No | No | No | No | No | No | Violation of IC3: No cancer diagnosis at last vaccination (baseline sample not evaluable); No booster vaccination on study (no other evaluable samples). |
| 0130 | Yes | Yes | No | NA | NA | NA |  |
| 0131 | Yes | Yes | No | NA | NA | No |  |
| 0132 | Yes | Yes | No | Yes | No | Yes |  |
| 0133 | Yes | Yes | No | NA | NA | Yes |  |
| 0134 | Yes | Yes | No | NA | NA | Yes |  |
| 0135 | Yes | Yes | No | NA | NA | NA |  |
| 0136 | No | No | Yes | No | No | No | Violation of IC3: No cancer diagnosis at last vaccination (baseline sample not evaluable); Cancer diagnosis at booster vaccination on study but remission <12months (no other evaluable samples). |
| 0137 | Yes | Yes | No | NA | NA | NA |  |
| 0138 | Yes | Yes | Yes | Yes | Yes | NA |  |
| 0139 | Yes | Yes | No | NA | NA | NA |  |
| 0140 | No | No | No | No | No | No | Violation of IC3: Diagnosis of active cancer at last vaccination but no active treatment (baseline sample not evaluable); No booster vaccination on study (no other samples evaluable). |
| 0141 | Yes | Yes | Yes | NA | Yes | NA |  |
| 0142 | Yes | Yes | Yes | Yes | Yes | NA |  |
| 0143 | Yes | Yes | Yes | Yes | NA | NA |  |
| 0144 | Yes | Yes | Yes | NA | Yes | NA |  |
| 0145 | Yes | Yes | No | NA | NA | NA |  |
| 0146 | Yes | Yes | No | NA | NA | NA |  |
| 0147 | Yes | Yes | Yes | NA | NA | NA |  |
| 0148 | No | No | No | No | No | No | Violation of IC3: Diagnosis of active cancer at last vaccination but no active treatment (baseline sample not evaluable); No booster vaccination on study (no other samples evaluable). |
| 0149 | Yes | Yes | Yes | NA | Yes | NA |  |
| 0150 | Yes | Yes | Yes | NA | NA | NA |  |
| 0151 | No | No | No | No | No | No |  |
| 0152 | Yes | Yes | No | NA | NA | NA |  |
| 0153 | Yes | Yes | Yes | NA | Yes | NA |  |

**Table legend:**

This table summarizes the evaluability of patients included in the I-SPARC trial across all study timepoints, including baseline, pre-booster, post-booster, and final assessment. Evaluability was assessed based on the availability of blood samples and adherence to protocol-defined eligibility criteria, including IC3 ("Cancer diagnosis, with active systemic treatment or in remission without treatment for ≥12 months, at last vaccination before enrolment" as per protocol version 6.0). Patients were categorized as evaluable or non-evaluable for each timepoint. Reasons for non-evaluability are detailed under the explanation column and include protocol deviations, absence of blood samples, changes in cancer treatment status, or lack of booster vaccination on study. This table provides a comprehensive overview of patient inclusion, retention, and data completeness throughout the study.

Abbreviations: EC, exclusion criteria; IC, Inclusion Criterion; NA, non-applicable.
